# Tailored Lattice “Tape” to Confine Tensile Interface for 11.08%‐Efficiency All‐Inorganic CsPbBr_3_ Perovskite Solar Cell with an Ultrahigh Voltage of 1.702 V

**DOI:** 10.1002/advs.202101418

**Published:** 2021-08-08

**Authors:** Qingwei Zhou, Jialong Duan, Jian Du, Qiyao Guo, Qiaoyu Zhang, Xiya Yang, Yanyan Duan, Qunwei Tang

**Affiliations:** ^1^ College of Information Science and Technology Jinan University Guangzhou 510632 P. R. China; ^2^ State Centre for International Cooperation on Designer Low‐Carbon and Environmental Material (SCICDLCEM) School of Materials Science and Engineering Zhengzhou University Zhengzhou 450001 P. R. China

**Keywords:** all‐inorganic CsPbBr_3_ perovskite solar cells, interface solidification, long‐term stability, strain release, Ti_3_C_2_ MXene

## Abstract

The crystal distortion such as lattice strain and defect located at the surfaces and grain boundaries induced by soft perovskite lattice highly determines the charge extraction‐transfer dynamics and recombination to cause an inferior efficiency of perovskite solar cells (PSCs). Herein, the authors propose a strategy to significantly reduce the superficial lattice tensile strain by means of incorporating an inorganic 2D Cl‐terminated Ti_3_C_2_ (Ti_3_C_2_Cl*_x_*) MXene into the bulk and surface of CsPbBr_3_ film. Arising from the strong interaction between Cl atoms in Ti_3_C_2_Cl*_x_* and the under‐coordinated Pb^2+^ in CsPbBr_3_ lattice, the expanded perovskite lattice is compressed and confined to act as a lattice “tape”, in which the Pb—Cl bond plays a role of “glue” and the 2D Ti_3_C_2_ immobilizes the lattice. Finally, the defective surface is healed and a champion efficiency as high as 11.08% with an ultrahigh open‐circuit voltage up to 1.702 V is achieved on the best all‐inorganic CsPbBr_3_ PSC, which is so far the highest efficiency record for this kind of PSCs. Furthermore, the unencapsulated device demonstrates nearly unchanged performance under 80% relative humidity over 100 days and 85 °C over 30 days.

## Graphical abstract

Arising from the formation of strong Pb—Cl bonding, chlorine terminated Ti_3_C_2_Cl*_x_* MXenes are used as lattice “tape” to reduce the defects and release tensile strain located at interfaces and grain boundaries of CsPbBr_3_ perovskite film, achieving a champion efficiency up to 11.08% with an ultrahigh voltage of 1.702 V for CsPbBr_3_ perovskite solar cells.

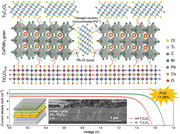

## 1 Introduction

Organic–inorganic hybrid perovskite solar cells (PSCs) have made a forward step towards upcoming commercialization due to the rapid increase of power conversion efficiency (PCE) from initial 3.8% to reported 25.6%.^[^ ^1^, ^2^, ^3^, ^4^, ^5^, ^6^ ^]^ However, the inherent decomposition of perovskite lattice under external stimulus such as moisture, oxygen, light and heat is still a stability burden in this way. Aiming to address this issue, all‐inorganic CsPb*X*_3_ (*X* = I^−^, Br^−^, or Cl^−^) perovskites have been gradually regarded as promising alternatives because of their superior environmental tolerances.^[^ ^7^, ^8^, ^9^, ^10^, ^11^, ^12^ ^]^ Among them, tri‐brominated CsPbBr_3_ perovskite presents the best weatherability regardless of their large bandgap around 2.3 eV comparing to I‐containing species,^[^ ^13^ ^]^ demonstrating a great potential in semitransparent photovoltaics or high‐voltage required electronics owing to the ultrahigh theoretical voltage output around 2.0 V.^[^ ^14^, ^15^, ^16^ ^]^ Up to date, the minimum PCE loss of inorganic CsPbBr_3_ PSC is still much higher than that of other devices because of the defective interfaces and grain boundaries induced open‐circuit voltage (*V*_oc_) deficit. As is well known, the soft feature of perovskite film allows for substantial distorted lattices during the phase conversion process under high temperature, which are mainly located at the superficial region and grain boundaries.^[^ ^17^, ^18^ ^]^ Apart from the popular positive under‐coordinated Pb^2+^ ions and negative Pb—*X* antisites (Pb*X*_3_^−^), the expanded or compressed lattice (in other words, tensile or compressive strain) in this area also determines the carrier transfer and ion migration.^[^ ^19^, ^20^ ^]^ How to heal the defective nanostructure and to realize the surface solidification undoubtedly maximize the device PCE of PSC.

Great efforts have been made to heal the perovskite surfaces by regulating the terminations.^[^ ^21^ ^]^ Inspired by the defect passivation in Si solar cells, many organic chemicals with functional groups such as pyridine, thiophene, and thiourea have been demonstrated to donate peripheral electrons to the under‐coordinated Pb^2+^ defects for an improved efficiency and stability based on Lewis acid‐base interaction.^[^ ^22^, ^23^ ^]^ Furthermore, the fabrication of “bone‐joint” configuration or soft layer by incorporating organic species with flexible chains can also effectively release the detrimental lattice strain.^[^ ^24^, ^25^ ^]^ However, the delicate character of organic molecules and their weak secondary bonding with perovskite lattice cause an inactivation under high temperature and longstanding light irradiation, which is incompatible with the all‐inorganic concept.^[^ ^26^ ^]^ Although inorganic passivation materials such as PbSO_4_, PbS, metal halides, and oxides are employed to increase the performance of PSCs,^[^ ^27^ ^]^ there is still no effective passivator to simultaneously reduce the above‐mentioned defects and control lattice strain. How to make defect‐free perovskite film by passivation‐engineering and strain‐engineering is of great importance to maximize the photovoltaic performances of all‐inorganic CsPbBr_3_ solar cells.

Arising from the strong electronic coupling, Cl atoms display superiority to thermodynamically stabilize the perovskite lattice and regulate the grain growth dynamics owing to the higher formation energy of Pb—Cl antisites at interface.^[^ ^28^ ^]^ But the prevalent Cl‐containing additives are always ionized, and the excess cations will destroy the perovskite lattice and induce unnecessary recombination centers. In this fashion, the cation size should be precisely controlled to avoid the size mismatch induced steric effect. As is well known, the newly emerging MXenes are new family members of the 2D transition‐metal carbides/nitrides with metallic conductivity and easily regulated termination with different chemical groups. Similar to graphene, the MXenes are obtained from the corresponding *MAX* (*M* = Ti, Nb, Mo, V, W, etc.; *X* = C or N; *A* = Al, Ga, Si, etc.) phases by selective etching A element and replacing the termination with F, Cl, OH, etc., commonly denoted as *T_x_*.^[^ ^29^ ^]^ Previous reports have demonstrated that the Ti_3_C_2_ *T_x_* MXene can effectively accelerate the charge extraction, enlarge grain size and increase the PCEs of PSCs.^[^ ^30^, ^31^ ^]^ However, it is still in lack of a better understanding on the lattice strain release and defect passivation for Ti_3_C_2_ *T_x_* MXene tailored perovskite films. Herein, we report an inorganic Cl‐terminated Ti_3_C_2_ (Ti_3_C_2_Cl*_x_*) MXene to fully heal the defective surfaces and grain boundaries of CsPbBr_3_ film. The best device achieves an efficiency as high as 11.08% with an ultrahigh *V*_oc_ of 1.702 V, to the best of our knowledge, these data are to date the highest PCE and *V*_oc_ records for the all‐inorganic CsPbBr_3_ PSCs. Apart from the strong passivation interaction between terminated Cl atoms and under‐coordinated Pb^2+^ ions and enlarged perovskite grain size, the bridging connection between Pb^2+^‐Cl‐Ti_3_C_2_ restricts the expansion of perovskite lattice to act as a lattice “tape” to reduce the tensile strain. As a result, the significantly reduced defect density in CsPbBr_3_ film allows for the stability advancement under persistent attack by 80% humidity or 85 °C in air for the device free of encapsulation.

## 2 Results and Discussion

**Figure 1:**
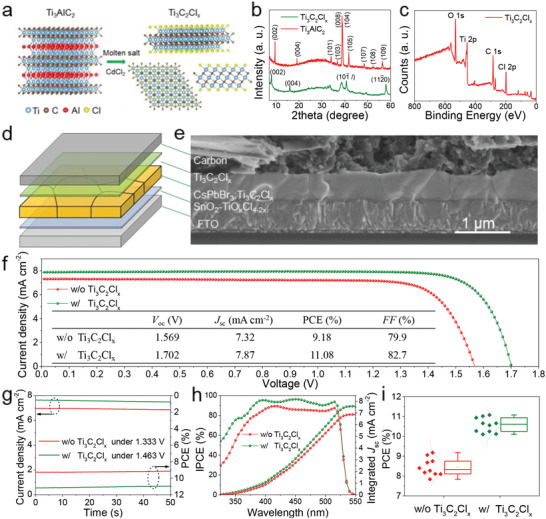
a) Schematic diagram of Ti_3_C_2_Cl*_x_* MXene preparation using Ti_3_AlC_2_ *MAX* in CdCl_2_ molten salt. b) XRD patterns of Ti_3_AlC_2_ *MAX* and Ti_3_C_2_Cl*_x_* MXene. c) XPS spectrum of Ti_3_C_2_Cl*_x_* MXene. d) The architecture and e) cross‐sectional SEM image of all‐inorganic PSC. f) *J–V* curves of control and optimized devices under reverse scan, g) steady power outputs, and h) IPCE spectra of PSCs. i) Statistical PCE distribution of pristine and optimal PSCs.

Cl‐terminated Ti_3_C_2_Cl*_x_* MXene is synthesized using a redox‐controlled Al etching of precursor Ti_3_AlC_2_ *MAX*‐phase in Lewis acidic CdCl_2_ melts combined with exfoliation and ultrasonication,^[^ ^32^ ^]^ the details can be found in the Supporting Information. As schematically shown in **Figure** **1a**, the unit cell of Ti_3_AlC_2_ *MAX*‐phase is composed of Ti_6_C octahedrons interleaved with layers of Al elements.^[^ ^33^ ^]^ Upon selectively removing Al atoms from interlayers with CdCl_2_ melts, an accordion‐like microstructure (Figure S1, Supporting Information) demonstrates the formation of 2D MXene, which can be cross‐checked by X‐ray diffraction (XRD) patterns.^[^ ^34^ ^]^ As shown in Figure 1b, most of the diffraction peaks of Ti_3_AlC_2_ *MAX*‐phase disappear in the final product, the characteristic diffraction peaks centered at 7.95°, 16.04°, 40.71°, and 57.98° correspond to the (002), (004), (10*l*), and (110) lattice planes of the Ti_3_C_2_ MXene,^[^ ^32^, ^35^ ^]^ respectively. Additionally, the disappearance of Al 2*p* signal and the appearance of a sharp peak of Cl 2*p* from X‐ray photoelectron spectroscopy (XPS) spectra (Figure 1c and Figures S2 and S3, Supporting Information) after etching treatment indicates the functional Cl termination on the Ti_3_C_2_Cl*_x_* MXene surface. The atomic ratio of Cl:Ti is determined to be 2:3 (11.69%:17.45%) based on the XPS results. The element mapping images (Figure S4, Supporting Information) also demonstrate the uniform distribution of Cl element on the surface of Ti_3_C_2_Cl*_x_* MXene. Through furiously mechanical liquid phase exfoliation, the delaminated Ti_3_C_2_Cl*_x_* MXene with thickness of ≈4 nm (Figure S5, Supporting Information) is obtained due to the weak interlayer van der Waals force. Arising from the ordered atom matrices with clear lattice fringes of defect‐free surface (Figure S6, Supporting Information), the as‐prepared Ti_3_C_2_Cl*_x_* MXene undoubtedly benefits the application in solar cells as lattice “tape” to regulate the chemical state of perovskite interfaces and grain boundaries.

Subsequently, all‐inorganic CsPbBr_3_ PSC with a configuration of FTO/SnO_2_‐TiO*_x_* Cl_4−2_ *_x_*/CsPbBr_3_:Ti_3_C_2_Cl*_x_*/ Ti_3_C_2_Cl*_x_*/carbon (Figure 1d) is fabricated by means of doping Ti_3_C_2_Cl*_x_* MXene into PbBr_2_ solution and spin‐coating Ti_3_C_2_Cl*_x_* onto the surface of final perovskite film. From the cross‐sectional scanning electron microscopy (SEM) image in Figure 1e, the device has a multilayered structure with a 60 nm SnO_2_‐TiO*_x_* Cl_4−2_ *_x_* layer, a 500 nm monolayer‐aligned perovskite overlayer and a 15 µm carbon electrode. By carefully optimizing the Ti_3_C_2_Cl*_x_* MXene dosage (Figure S7 and Table S1, Supporting Information), a champion PCE as high as 11.08% with an ultrahigh *V*_oc_ of 1.702 V, a short‐circuit current density (*J*_sc_) of 7.87 mA cm^−2^, and a fill factor (*FF*) of 82.7% has been achieved by recording the characteristic photocurrent–voltage (*J–V*) curves via reverse scan under standard AM 1.5 G illumination, as shown in Figure 1f, which is much higher than that of pristine device with 9.18%‐efficiency. All the increased photovoltaic data including *V*_oc_, *J*_sc_, and *FF* are attributed to the reduction of charge recombination after adding Cl‐terminated Ti_3_C_2_ MXene.^[^ ^36^ ^]^ To the best of our knowledge, both the PCE of 11.08% and *V*_oc_ of 1.702 V are till now the highest records for state‐of‐the‐art all‐inorganic CsPbBr_3_ PSCs (Table S2, Supporting Information). The steady power outputs of inorganic CsPbBr_3_ PSCs under bias voltage at maximum power point (Figure 1g) and incident photon‐to‐current efficiency (IPCE) spectra (Figure 1h) are characterized to cross‐check the validity of PCE enhancement. A much higher steady efficiency of 11.0% and an increased integrated current density of 7.59 mA cm^−2^ for Ti_3_C_2_Cl*_x_*‐tailored CsPbBr_3_ PSC from 6.90 mA cm^−2^ for control device demonstrate the validity of the above‐mentioned conclusion, highly agreeing well with the *J–V* measurements. Moreover, the hysteresis factor of devices has been reduced from 21.6% to 14.6% after the modification (Figure S8 and Table S3, Supporting Information). The statistical photovoltaic data of random 10 individual devices with and without Ti_3_C_2_Cl*_x_* modification also displays an obvious increase in PCE (Figure 1i) and other parameters (Figure S9, Supporting Information), further suggesting the positive effect of Ti_3_C_2_Cl*_x_* on solar‐to‐electric conversion. To highlight the superiority of Ti_3_C_2_ MXene in PSCs, we have also prepared F‐terminated Ti_3_C_2_F*_x_* and Br‐terminated Ti_3_C_2_Br*_x_* MXenes (Figure S10, Supporting Information) and a similar PCE enhancement is obtained after optimized the blending ratio of MXene (the optimized concentration is controlled at 0.02 mg mL^−1^, see Figure S11 and Table S4, Supporting Information). Taking the labile surface bonding of Cl/Br‐terminated MXene into consideration, versatile synthons for further chemical transformations provide multi‐possibilities to heal the defective surface of CsPbBr_3_ perovskite.

**Figure 2:**
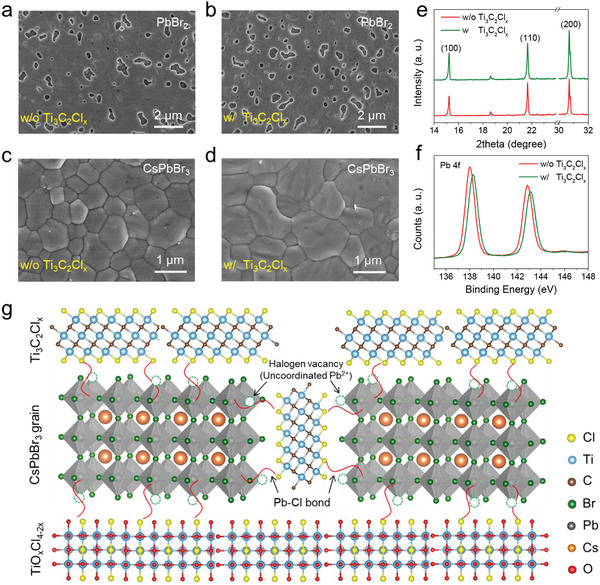
Top‐view SEM images of PbBr_2_ films and CsPbBr_3_ perovskite films a,c) without and b,d) with Ti_3_C_2_Cl*_x_* additive. e) XRD patterns and f) XPS spectra of Pb 4*f* for CsPbBr_3_ perovskite films. g) Schematic diagram of full defect passivation in CsPbBr_3_ film by Ti_3_C_2_Cl*_x_* MXene.

To reveal the intrinsic mechanism behind the efficiency enhancement, we first explore the effect of Ti_3_C_2_Cl*_x_* on the film quality. As shown in **Figure** **2a**,b and Figure S12, Supporting Information, the Ti_3_C_2_Cl*_x_*‐tailored PbBr_2_ film with larger porosity is formed during the solvent volatilization.^[^ ^37^ ^]^ These nanoholes undoubtedly provide enough pathways for subsequent CsBr diffusion and match pre‐expanded volume for perovskite grain growth,^[^ ^38^ ^]^ helping make a high‐quality CsPbBr_3_ film with enlarged grain size and compact grain boundary (Figure 2c,d), which is accordance with the increased XRD peak intensities (Figure 2e) and enhanced light absorption ability (Figures S13 and S14, Supporting Information). The mechanism behind the regulated film growth dynamics is attributed to the stronger electronic coupling between Pb and Cl atoms (Figure S15, Supporting Information) than that with Br atoms. Upon introducing the Ti_3_C_2_Cl*_x_* into the perovskite precursor, partial PbBr_2_ lattice will bond with the dangling Cl atoms in Ti_3_C_2_Cl*_x_* to spatially retard the nucleation around the additives.^[^ ^39^, ^40^ ^]^ In the following phase conversion process from PbBr_2_ to CsPbBr_3_, the homogeneously distributed Ti_3_C_2_Cl*_x_* additives (Figures S16 and S17, Supporting Information) will play a role of “reservoir” to slow down the intercalation of Cs^+^ into underlying PbBr_2_ frameworks. Furthermore, the strong electron‐withdrawing properties of Cl atoms cause the binding energy of Pb 4*f* to shift up by 0.26 eV for Ti_3_C_2_Cl*_x_*‐tailored CsPbBr_3_ (Figure 2f),^[^ ^41^ ^]^ in other words, the electron cloud over under‐coordinated Pb^2+^ defect will be efficiently delocalized and redistributed over the whole lattice evenly, which in turn regulates the binding energies of Cs and Br elements (Figure S18, Supporting Information).^[^ ^42^, ^43^ ^]^ Similarly, the slight shifts of Cs 3*d*, Pb 4*f*, and Br 3*d* for the case of only Ti_3_C_2_Cl*_x_* surface modification also indicate the formation of Pb—Cl bonds at the CsPbBr_3_/Ti_3_C_2_Cl*_x_* interface (Figure S19, Supporting Information). Combined with the current results, we propose a concept of full defect passivation strategy here, as illustrated in Figure 2g, the strong binding interaction between under‐coordinated Pb^2+^ ions (V*_X_*) and Cl atoms is responsible for the reduction of halide ions deficiency or excess induced defects due to the higher formation energy of Pb—Cl antisites at interfaces,.^[^ ^44^ ^]^ It is worthy to mention that the underlying surface of perovskite film is passivated by the electron transfer layer SnO_2_/TiO*_x_* Cl_4−2_ *_x_* according to our previous report.^[^ ^45^ ^]^

**Figure 3:**
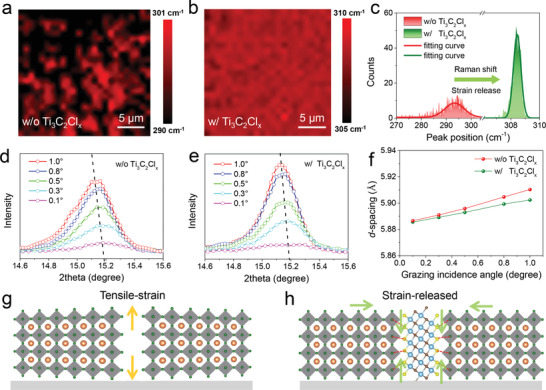
Raman mapping images of CsPbBr_3_ perovskite films a) without and b) with Ti_3_C_2_Cl*_x_* MXene, and c) the corresponding distribution statistics of Raman peak for different perovskite films. GIXRD patterns of CsPbBr_3_ (100) plane for the perovskite films d) without and e) with Ti_3_C_2_Cl*_x_* modification. f) *d*‐spacing values obtained from the GIXRD patterns as a function of the incidence angle. g) Schematic diagram of residual strain in the pristine CsPbBr_3_ grains, and h) schematic diagram of released strain in the reinforced CsPbBr_3_ grains with Ti_3_C_2_Cl*_x_* MXene.

The surface lattices suffer from distortion to cause the formation of residual stress in perovskite film owing to the soft feature of perovskite, which is detrimental to device performance. Taking the stronger binding energy of Pb—Cl (301 kJ mol^−1^) in comparison to 248 kJ mol^−1^ for Pb—Br into consideration, the Cl terminated Ti_3_C_2_ MXene can drag the adjacent perovskite grains closer (reduced grain gap as shown in Figure 2d), anchoring the Pb atoms and confining the perovskite lattice without deformation owing to the immovable 2D Ti_3_C_2_ substrate. Following this line of thoughts, the Ti_3_C_2_Cl*_x_* is expected to be surface lattice “tape” to hinder the lattice expansion or contraction and reduce the residual stress. To deeply understand the strain evolution, we have characterized the Raman mapping images in **Figure** **3a**,b. In theory, the vibrational mode around 309 cm^−1^ in Raman spectra presents the reorientation of Cs^+^ in CsPbBr_3_ lattice influenced by the PbBr_6_ octahedral framework, which is strongly dependent on the lattice strain.^[^ ^46^, ^47^ ^]^ The enlarged lattice allows for a blue shift of Raman peak under the presence of tensile strain.^[^ ^48^ ^]^ Therefore, the pristine CsPbBr_3_ perovskite film suffers from a serious tensile strain owing to the lower average wavenumber around 295 cm^−1^ for Cs^+^ vibrational mode in Figure 3c, which is mainly formed during the annealing process owing to the different thermal expansion coefficients of adjacent layers in the PSC devices.^[^ ^49^ ^]^ Higher tensile strain results in more serious lattice distortion, larger charge transfer barrier, and lower ions migration energy.^[^ ^50^ ^]^ Upon introducing Ti_3_C_2_Cl*_x_* MXene into the CsPbBr_3_ film, homogeneously distributed Raman peak for Cs^+^ vibrational mode is closer to 309 cm^−1^ (Figure 3b,c), this is a clear indicator of the released tensile strain in the perovskite film. We have further investigated the strain distribution by depth‐dependent grazing incident X‐ray diffraction (GIXRD) measurement to better highlight the lattice distortion in CsPbBr_3_ films. As shown in Figure 3d and Figure S20, Supporting Information, the obvious shift of the characteristic peaks for pristine perovskite film to lower angles along with increasing the incidence angle demonstrates gradually increased crystal plane distance along the perpendicular direction to substrate according to Bragg's Lawall, in other words, the pristine film suffers from a serious tensile strain. In contrast, the CsPbBr_3_ perovskite film with Ti_3_C_2_Cl*_x_* modification delivers a significantly reduced shift of diffraction peaks (Figure 3e and Figure S20, Supporting Information). The plots of *d*‐spacing values for (100) planes as a function of incident angle demonstrate the lattices across whole film especially in bottom region are contracted, as shown in Figure 3f. Owing to the strong electron‐withdrawing property of terminated Cl atoms to bond with under‐coordinated Pb^2+^ ion in the perovskite, 2D Ti_3_C_2_Cl*_x_* MXene can tightly adhere to the surface of CsPbBr_3_ grains, like a lattice “tape” to heal the soft perovskite lattice and to relieve the lattice expansion (Figure 3g,h).

**Figure 4:**
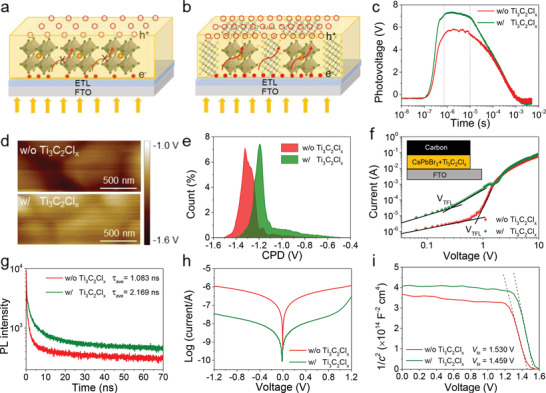
Illustration for the photogenerated carrier transfer in a) pristine and b) Ti_3_C_2_Cl*_x_* containing perovskite films. c) TSPV curves of various PSCs. d) KPFM images and e) the corresponding CPD distributions of perovskite films. f) Dark *J–V* curves for the hole‐only devices with and without Ti_3_C_2_Cl*_x_* MXene. g) TRPL curves of perovskite films with and without Ti_3_C_2_Cl*_x_*. h) Dark *J–V* curves and i) Mott–Schottky curves of various PSCs.

Under light irradiation, the defects and strain in perovskite film will undoubtedly cause a carrier loss during the transfer process, as illustrated in **Figure** **4a**, leaving less holes accumulated on the perovskite top surface. In this fashion, there will be more holes to call for a larger photovoltage upon healing and solidifying the defective surfaces and grain boundaries (Figure 4b). Aiming to visually distinguish the surface charge, we have further carried out transient surface photovoltage (TSPV) measurement and the schematic setup is provided in Figure S21, Supporting Information. As shown in Figure 4c, the higher positive signal of 7.36 V under pulse laser stimuli for Ti_3_C_2_Cl*_x_*‐tailored perovskite film suggests an increased hole concentration,^[^ ^51^ ^]^ which can be cross‐checked by the higher contact potential difference (CPD) than that of pristine perovskite film obtained from Kelvin probe force microscopy (KPFM) images (Figure 4d,e), therefore, the defects located at skin layer induced by dangling bonds and tensile strain are healed. We then quantitatively evaluated the defect‐state density (*n*_t_) in the perovskite films using a hole‐only device according to the space‐charge‐limited‐current (SCLC) model.^[^ ^52^ ^]^ As shown in Figure 4f, the reduced trap‐filled limit voltage (*V*_TFL_) from 0.78 to 0.56 V for Ti_3_C_2_Cl*_x_* passivated perovskite film means a significantly reduced *n*_t_ from 5.93 × 10^15^ to 1.92 × 10^15^ cm^−3^, which can be cross‐checked by the increased photoluminescence (PL) peak intensity (Figure S22, Supporting Information) because PL behavior of perovskite film is highly dependent on the defect density.^[^ ^53^ ^]^ When assembled into a PSC, the nonradiative recombination is suppressed to produce a prolonged lifetime from 1.083 to 2.169 ns (Figure 4g and Table S5, Supporting Information) for a voltage and efficiency enhancement. As shown in Figure 4h, the reverse current density of optimized device under dark condition is over one order of magnitude lower than that of reference device, demonstrating that more photogenerated carriers pass through the device rather than direct shunting along with the defect‐assisted channels.^[^ ^54^ ^]^ By further recording capacitance–voltage curves in the dark according to the Mott–Schottky relationship (Figure 4i), a higher built‐in electric field (*V*_bi_) for the Ti_3_C_2_Cl*_x_*‐tailored PSC implies an enhanced driving force for charge separation (improved electron lifetime, see Figure S23, Supporting Information) and an extended depletion region for suppressing charge recombination (enlarged recombination resistance, see Figure S24, Supporting Information),^[^ ^55^, ^56^ ^]^ which is one main origin for the ultrahigh *V*_oc_. Apart from the reduced defect and the released strain, we have further characterized the band structure of MXene. As shown in Figure S25, Supporting Information, the work function of Ti_3_C_2_Cl*_x_* Mxene obtained from ultraviolet photoelectron spectroscopy (UPS) is deterimined to be −4.62 eV, which will upward bend the valence and conduction band of CsPbBr_3_ at the interface, accelerating the charge extraction.^[^ ^57^ ^]^

**Figure 5:**
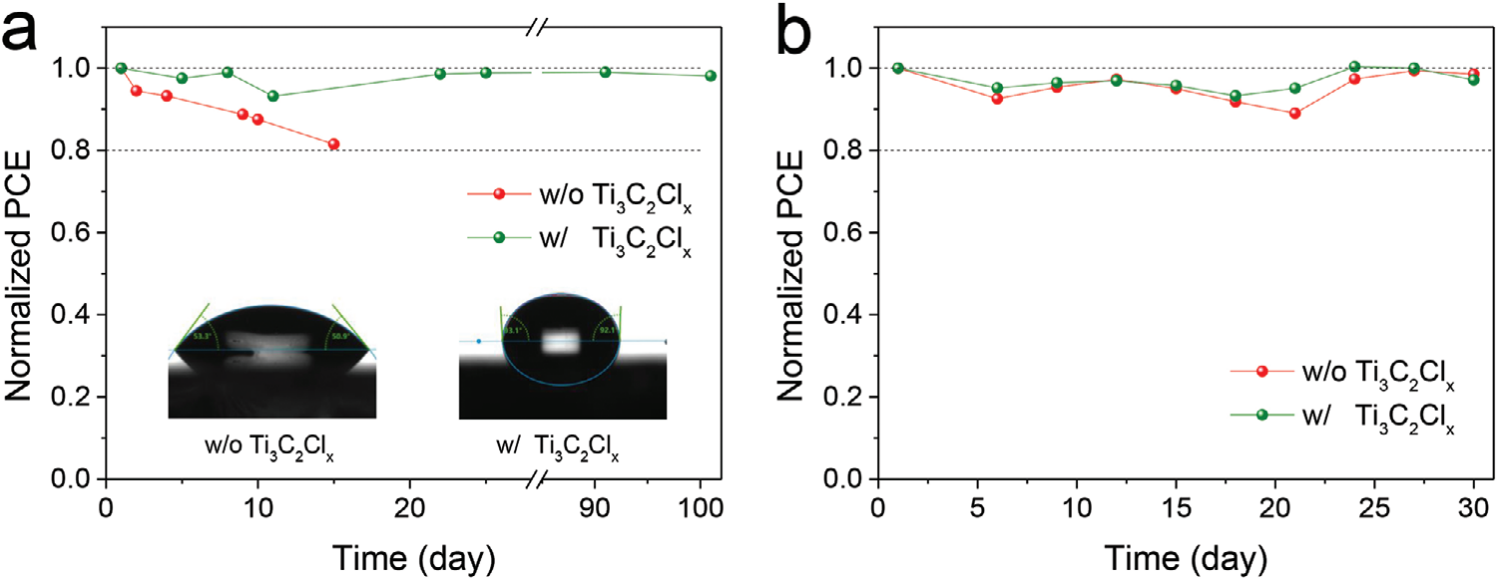
Long‐term stability of the encapsulation‐free devices with and without Ti_3_C_2_Cl*_x_* in air under a) 25 °C, 80% RH and b) 85 °C, 40% RH conditions.

Finally, the long‐term stability of all‐inorganic CsPbBr_3_ PSCs is evaluated under persistent moisture or thermal attacks. As shown in **Figure** **5a**, the Ti_3_C_2_Cl*_x_*‐repaired PSC device free of encapsulation delivers a stabilized PCE performance under 25 °C and 80% relative humidity (RH) in air over 100 days, which is mainly attributed to the reduced defect and contracted lattice to trigger unwanted phase attenuation and the protection of hydrophobic Ti_3_C_2_Cl*_x_* encapsulant (enlarged contact angle from 50.9° to 92.1°).^[^ ^58^ ^]^ Owing to the absence of damageable organic species, both two PSCs present excellent thermal stability. As shown in Figure 5b, there is nearly no PCE degradation after 30 days storage under 85 °C, 40% RH condition, demonstrating the advantages of functionalized Ti_3_C_2_ as lattice “tape” to enhance the performance of highly‐efficient PSCs. It is worthy to note that the performance improvement behavior of PSC device in stability test is related to the film quality improvement after storage for a few days under controlled ambient atmosphere.^[^ ^59^ ^]^

## 3 Conclusions

In summary, we have demonstrated a novel strategy to simultaneously realize defect passivation and strain release by incorporating Cl‐terminated Ti_3_C_2_ MXene into the bulk and surface of perovskite film for stable and efficient all‐inorganic CsPbBr_3_ PSCs. Arising from the formation of Pb—Cl bond between the interfacial under‐coordinated Pb^2+^ ions in perovskite lattice and terminated Cl atoms in Ti_3_C_2_Cl*_x_* MXene, the defects and expanded lattices located at the interfaces and grain boundaries in perovskite film are significantly healed, playing a role of surface lattice “tape”, in which the Pb—Cl bond represents “glue” and Ti_3_C_2_ immobilizes the lattice. As a result, the best all‐inorganic CsPbBr_3_ PSC achieves an efficiency as high as 11.08% with an ultrahigh *V*_oc_ of 1.702 V, which are the highest PCE and voltage records for CsPbBr_3_ solar cells. Moreover, the unencapsulated solar cell presents an excellent stability under high humidity (80% RH) and high temperature (85 °C) conditions over 100 and 30 days, respectively. Our findings open up a new avenue for preparing high‐quality defect‐free perovskite films, which not only benefits preparing high‐performance PSCs but also other optoelectronic devices based on perovskites.

## 4 Experimental Section

### Materials and Reagents

SnCl_2_·2H_2_O (Alfa Aesar), CH_4_N_2_S (Macklin), TiCl_4_ (Aladdin), Ti_3_AlC_2_ (Mianyang Hengchuan Electrical Equipment Sales Co., Ltd.), PbBr_2_ (Aladdin), CsBr (Aladdin), *N*,*N*‐dimethylformamide (DMF, Sinopharm), CH_3_OH (Sinopharm), isopropanol (IPA, Aladdin), HCl (Guangzhou Chemical Reagent Factory), FTO glass (12 Ω per square), Zn powder (Guangzhou Chemical Reagent Factory), carbon paste (Shanghai MaterWin New Materials Co., Ltd). All materials were used as supplied without further treatment.

### Synthesis of Ti_3_C_2_Cl*_x_* MXene

The Cl‐terminated Ti_3_C_2_ MXene was fabricated through molten salt‐based etching of Ti_3_AlC_2_ *MAX* phase and surface group substitution/elimination reactions in a N_2_‐filled glovebox.^[^ ^35^ ^]^ 0.5 g of Ti_3_AlC_2_ was first mixed with 3.768 g of CdCl_2_ (molar ratio of 1:8). The resultant mixture was then heated in an alumina crucible at 610 °C for 7 h, which involved the following reactions.

(1) $$2Ti_{3}\mathrm{Al}C_{2}+3\mathrm{CdC}l_{2}\to 2Ti_{3}C_{2}+2\mathrm{AlC}l_{3}+3\mathrm{Cd}$$

(2) $$Ti_{3}C_{2}+\mathrm{CdC}l_{2}\to Ti_{3}C_{2}Cl_{2}+\mathrm{Cd}$$

After cooling to room temperature, the powders were washed by concentrated aqueous HCl (12.1 m) to remove excess CdCl_2_ and metallic Cd, followed by washing with deionized water for several times until pH ≈ 6.5. The resultant Cl functionalized MXene powders were dried under vacuum at 45 °C. Finally, by liquid phase exfoliation of Cl functionalized MXene powders in IPA, the 2D few‐layer Cl‐terminated Ti_3_C_2_Cl*_x_* MXene could be obtained. In details, 0.1 g of Cl functionalized MXene powders was added to 50 mL of IPA and then ultrasonicated for 25 h at a power of 300 W. The upper dark green suspension was obtained after centrifuging at 10 000 rpm for 30 min to separate the un‐exfoliated MXene. Before usage, the obtained Ti_3_C_2_Cl*_x_* MXene suspension was filtered with 0.22 µm PTFE filter.

### Solar Cell Fabrication

The FTO‐coated glass was etched with Zn powders and HCl aqueous solution to obtain the desired pattern and then ultrasonic cleaned with detergent, deionized water, and ethanol. The cleaned FTO substrates were further treated by plasma for 5 min. Before deposition, FTO substrates and SnO_2_ QDs solution were preheated at 80 °C, in which SnO_2_ QDs solution was prepared according to our previous reports.^[^ ^45^ ^]^ Compact SnO_2_ layer was deposited onto FTO substrate by spin‐coating at 2000 rpm for 30 s and subsequently annealed at 200 °C for 1 h in air. Then, TiCl_4_ treatment was performed by soaking the SnO_2_ coated FTO glass in TiCl_4_ aqueous solution (40 mm) at 75 °C for 30 min. After washed with deionized water and ethanol, the substrate was then annealed in air at 200 °C for 30 min. The CsPbBr_3_ film was fabricated by a multi‐step spin‐coating method. In details, 1 m PbBr_2_ DMF solution with different amount of Ti_3_C_2_Cl*_x_* was spin‐coated on the substrate at 2000 rpm for 30 s under 100 °C and then dried at 100 °C for 30 min. After that, 0.07 m CsBr methanol solution was spin‐coated onto PbBr_2_ film at 2000 rpm for 30 s, and heated at 250 °C for 5 min. By repeated this process for several times, a high quality CsPbBr_3_ film was obtained. 1 mg mL^−1^ Ti_3_C_2_Cl*_x_* suspension in IPA was spin‐coated on the surface of CsPbBr_3_ film at 2000 rpm for 30 s, and then annealed in air at 100 °C for another 5 min. Finally, a conductive carbon electrode with active area of 0.09 cm^2^ was coated on the perovskite film by a blade coating method.

### Characterizations

The morphologies were obtained by a field emission SEM (Hitachi S4800) and transmission electron microscope (Tecnai G2 F20). The element analysis was carried out using SEM (FEI QUANTA250 and Zeiss Gemini300). The Raman mapping was recorded using an InVia Raman system (a42K864 Renishaw, InVia system) with an excitation laser wavelength of 532 nm. The film thickness of MXene was obtained by atomic force microscopy (AFM, Seiko SPA400). The surface potential was characterized by KPFM (Multimode 8, Bruker, German). The XRD and GIXRD patterns of perovskite and MXenes were conducted on an X‐ray diffractometer (Bruker D8 Advance). PL spectra were recorded via a fluorescence spectrometer excited by a 350 nm laser and the TRPL spectra were characterized using a Horiba spectrometer with an excitation wavelength of 500 nm. The characteristic *J–V* curves of PSCs were recorded on an electrochemical workstation (CHI660E) under irradiation of simulated solar light (Newport, Oriel Class 3A, 91195A). The light intensity was controlled at 100 mW cm^−2^ calibrated by a standard silicon solar cell. The IPCE spectra of various devices were obtained using an IPCE kit from Enli Technology Co., Ltd. The *V*_oc_ decay curves were measured at open‐circuit mode by illuminating the device for several seconds, and then instantaneously switching off the light. The TSPV signals were recorded by a home‐made setup using a 355 nm laser. Capacitance–voltage (*C–V*) curves were measured at a frequency of 5 kHz with an amplitude of 5 mV under dark. Tafel curves of various devices were measured on CHI660E electrochemical workstation.

## Conflict of Interest

The authors declare no conflict of interest.

## Supporting information

Supporting Information

## Data Availability

Data sharing is not applicable to this article as no new data were created or analyzed in this study.

## References

[1] A. Kojima , K. Teshima , Y. Shirai , T. Miyasaka , J. Am. Chem. Soc. 2009, 131, 6050. 19366264 10.1021/ja809598r

[2] H. Chen , F. Ye , W. Tang , J. He , M. Yin , Y. Wang , F. Xie , E. Bi , X. Yang , M. Grätzel , L. Han , Nature 2017, 550, 92. 28869967 10.1038/nature23877

[3] Y. Wang , M. I. Dar , L. K. Ono , T. Zhang , M. Kan , Y. Li , L. Zhang , X. Wang , Y. Yang , X. Gao , Y. Qi , M. Grätzel , Y. Zhao , Science 2019, 365, 591. 31395783 10.1126/science.aav8680

[4] Y. Xu , M. Wang , Y. Lei , Z. Ci , Z. Jin , Adv. Energy Mater. 2020, 10, 2002558.

[5] J. Peng , D. Walter , Y. Ren , M. Tebyetekerwa , Y. Wu , T. Duong , Q. Lin , J. Li , T. Lu , M. A. Mahmud , O. L. C. Lem , S. Zhao , W. Liu , Y. Liu , H. Shen , L. Li , F. Kremer , H. T. Nguyen , D.‐Y. Choi , K. J. Weber , K. R. Catchpole , T. P. White , Science 2021, 371, 390. 33479151 10.1126/science.abb8687

[6] J. Jeong , M. Kim , J. Seo , H. Lu , P. Ahlawat , A. Mishra , Y. Yang , M. A. Hope , F. T. Eickemeyer , M. Kim , Y. J. Yoon , I. W. Choi , B. P. Darwich , S. J. Choi , Y. Jo , J. H. Lee , B. Walker , S. M. Zakeeruddin , L. Emsley , U. Rothlisberger , A. Hagfeldt , D. S. Kim , M. Grätzel , J. Y. Kim , Nature 2021, 592, 381. 33820983 10.1038/s41586-021-03406-5

[7] J. Duan , Y. Zhao , B. He , Q. Tang , Angew. Chem., Int. Ed. 2018, 57, 3787. 10.1002/anie.201800019 29380514

[8] Q. Wang , Z. Jin , D. Chen , D. Bai , H. Bian , J. Sun , G. Zhu , G. Wang , S. F. Liu , Adv. Energy Mater. 2018, 8, 1800007.

[9] W. Zhu , Z. Zhang , W. Chai , Q. Zhang , D. Chen , Z. Lin , J. Chang , J. Zhang , C. Zhang , Y. Hao , ChemSusChem 2019, 12, 2318. 30912615 10.1002/cssc.201900611

[10] L. Yan , Q. Xue , M. Liu , Z. Zhu , J. Tian , Z. Li , Z. Chen , Z. Chen , H. Yan , H.‐L. Yip , Y. Cao , Adv. Mater. 2018, 30, 1802509. 10.1002/adma.201802509 29971864

[11] H. Bian , D. Bai , Z. Jin , K. Wang , L. Liang , H. Wang , J. Zhang , Q. Wang , S. (Frank) Liu , Joule 2018, 2, 1500.

[12] Z. Li , F. Zhou , H. Yao , Z. Ci , Z. Yang , Z. Jin , Mater. Today 2021, 10.1016/j.mattod.2021.01.028.

[13] J. Zhang , G. Hodes , Z. Jin , S. F. Liu , Angew. Chem., Int. Ed. 2019, 58, 15596. 10.1002/anie.201901081 30861267

[14] J. Liang , C. Wang , Y. Wang , Z. Xu , Z. Lu , Y. Ma , H. Zhu , Y. Hu , C. Xiao , X. Yi , G. Zhu , H. Lv , L. Ma , T. Chen , Z. Tie , Z. Jin , J. Liu , J. Am. Chem. Soc. 2016, 138, 15829. 27960305 10.1021/jacs.6b10227

[15] J. Duan , T. Hu , Y. Zhao , B. He , Q. Tang , Angew. Chem., Int. Ed. 2018, 57, 5746. 10.1002/anie.201801837 29603834

[16] D. Huang , P. Xie , Z. Pan , H. Rao , X. Zhong , J. Mater. Chem. A 2019, 7, 22420.

[17] Y. Lin , Y. Liu , S. Chen , S. Wang , Z. Ni , C. H. Van Brackle , S. Yang , J. Zhao , Z. Yu , X. Dai , Q. Wang , Y. Deng , J. Huang , Energy Environ. Sci. 2021, 14, 1563.

[18] W. Chu , W. A. Saidi , J. Zhao , O. V. Prezhdo , Angew. Chem., Int. Ed. 2020, 59, 6435. 10.1002/anie.201915702 31958363

[19] C. Zhu , X. Niu , Y. Fu , N. Li , C. Hu , Y. Chen , X. He , G. Na , P. Liu , H. Zai , Y. Ge , Y. Lu , X. Ke , Y. Bai , S. Yang , P. Chen , Y. Li , M. Sui , L. Zhang , H. Zhou , Q. Chen , Nat. Commun. 2019, 10, 815. 30778061 10.1038/s41467-019-08507-4 PMC6379394

[20] D.‐J. Xue , Y. Hou , S.‐C. Liu , M. Wei , B. Chen , Z. Huang , Z. Li , B. Sun , A. H. Proppe , Y. Dong , M. I. Saidaminov , S. O. Kelley , J.‐S. Hu , E. H. Sargent , Nat. Commun. 2020, 11, 1514. 32251277 10.1038/s41467-020-15338-1 PMC7090003

[21] S. Bi , X. Leng , Y. Li , Z. Zheng , X. Zhang , Y. Zhang , H. Zhou , Adv. Mater. 2019, 31, 1805708. 10.1002/adma.201805708 30600552

[22] B. Chen , P. N. Rudd , S. Yang , Y. Yuan , J. Huang , Chem. Soc. Rev. 2019, 48, 3842. 31187791 10.1039/c8cs00853a

[23] F. Gao , Y. Zhao , X. Zhang , J. You , Adv. Energy Mater. 2020, 10, 1902650.

[24] H. Wang , C. Zhu , L. Liu , S. Ma , P. Liu , J. Wu , C. Shi , Q. Du , Y. Hao , S. Xiang , H. Chen , P. Chen , Y. Bai , H. Zhou , Y. Li , Q. Chen , Adv. Mater. 2019, 31, 1904408. 10.1002/adma.201904408 31617644

[25] X. Meng , Z. Cai , Y. Zhang , X. Hu , Z. Xing , Z. Huang , Z. Huang , Y. Cui , T. Hu , M. Su , X. Liao , L. Zhang , F. Wang , Y. Song , Y. Chen , Nat. Commun. 2020, 11, 3016. 32541859 10.1038/s41467-020-16831-3 PMC7295992

[26] H. Zhang , Y. Wu , C. Shen , E. Li , C. Yan , W. Zhang , H. Tian , L. Han , W. Zhu , Adv. Energy Mater. 2019, 9, 1803573.

[27] W. Qi , X. Zhou , J. Li , J. Cheng , Y. Li , M. J. Ko , Y. Zhao , X. Zhang , Sci. Bull. 2020, 65, 2022. 10.1016/j.scib.2020.07.017 36659061

[28] P. Zhu , S. Gu , X. Luo , Y. Gao , S. Li , J. Zhu , H. Tan , Adv. Energy Mater. 2019, 10, 1903083.

[29] M. Naguib , O. Mashtalir , J. Carle , V. Presser , J. Lu , L. Hultman , Y. Gogotsi , M. W. Barsoum , ACS Nano 2012, 6, 1322. 22279971 10.1021/nn204153h

[30] L. Yang , C. Dall'Agnese , Y. Dall'Agnese , G. Chen , Y. Gao , Y. Sanehira , A. K. Jena , X. Wang , Y. Gogotsi , T. Miyasaka , Adv. Funct. Mater. 2019, 29, 1905694.

[31] X. Chen , W. Xu , N. Ding , Y. Ji , G. Pan , J. Zhu , D. Zhou , Y. Wu , C. Chen , H. Song , Adv. Funct. Mater. 2020, 30, 2003295.

[32] Y. Li , H. Shao , Z. Lin , J. Lu , L. Liu , B. Duployer , P. O. Å. Persson , P. Eklund , L. Hultman , M. Li , K. Chen , X.‐H. Zha , S. Du , P. Rozier , Z. Chai , E. Raymundo‐Piñero , P.‐L. Taberna , P. Simon , Q. Huang , Nat. Mater. 2020, 19, 894. 32284597 10.1038/s41563-020-0657-0

[33] M. Naguib , M. Kurtoglu , V. Presser , J. Lu , J. Niu , M. Heon , L. Hultman , Y. Gogotsi , M. W. Barsoum , Adv. Mater. 2011, 23, 4248. 21861270 10.1002/adma.201102306

[34] M. Li , J. Lu , K. Luo , Y. Li , K. Chang , K. Chen , J. Zhou , J. Rosen , L. Hultman , P. Eklund , P. O. Å. Persson , S. Du , Z. Chai , Z. Huang , Q. Huang , J. Am. Chem. Soc. 2019, 141, 4730. 30821963 10.1021/jacs.9b00574

[35] V. Kamysbayev , A. S. Filatov , H. Hu , X. Rui , F. Lagunas , D. Wang , R. F. Klie , D. V. Talapin , Science 2020, 369, 979. 32616671 10.1126/science.aba8311

[36] N. H. Tiep , Z. Ku , H. J. Fan , Adv. Energy Mater. 2016, 6, 1501420.

[37] L. Zhu , J. Shi , S. Lv , Y. Yang , X. Xu , Y. Xu , J. Xiao , H. Wu , Y. Luo , D. Li , Q. Meng , Nano Energy 2015, 15, 540.

[38] Y. Zhao , J. Duan , Y. Wang , X. Yang , Q. Tang , Nano Energy 2020, 67, 104286.

[39] S.‐S. Li , C.‐H. Chang , Y.‐C. Wang , C.‐W. Lin , D.‐Y. Wang , J.‐C. Lin , C.‐C. Chen , H.‐S. Sheu , H.‐C. Chia , W.‐R. Wu , U.‐S. Jeng , C.‐T. Liang , R. Sankar , F.‐C. Chou , C.‐W. Chen , Energy Environ. Sci. 2016, 9, 1282.

[40] Z. Guo , L. Gao , Z. Xu , S. Teo , C. Zhang , Y. Kamata , S. Hayase , T. Ma , Small 2018, 14, 1802738. 10.1002/smll.201802738 30300503

[41] Y. Wang , T. Wu , J. Barbaud , W. Kong , D. Cui , H. Chen , X. Yang , L. Han , Science 2019, 365, 687. 31416961 10.1126/science.aax8018

[42] J. K. Nam , S. U. Chai , W. Cha , Y. J. Choi , W. Kim , M. S. Jung , J. Kwon , D. Kim , J. H. Park , Nano Lett. 2017, 17, 2028. 28170276 10.1021/acs.nanolett.7b00050

[43] Y. Li , J. Duan , H. Yuan , Y. Zhao , B. He , Q. Tang , Sol. RRL 2018, 2, 1800164.

[44] Y. Wang , G. Chen , D. Ouyang , X. He , C. Li , R. Ma , W. Yin , W. C. H. Choy , Adv. Mater. 2020, 32, 2000186. 10.1002/adma.202000186 32363655

[45] Q. Zhou , J. Duan , Y. Wang , X. Yang , Q. Tang , J. Energy Chem. 2020, 50, 1.

[46] J.‐H. Cha , J. H. Han , W. Yin , C. Park , Y. Park , T. K. Ahn , J. H. Cho , D.‐Y. Jung , J. Phys. Chem. Lett. 2017, 8, 565. 28067051 10.1021/acs.jpclett.6b02763

[47] J. Ibaceta‐Jaña , R. Muydinov , P. Rosado , H. Mirhosseini , M. Chugh , O. Nazarenko , D. N. Dirin , D. Heinrich , M. R. Wagner , T. D. Kühne , B. Szyszka , M. V. Kovalenko , A. Hoffmann , Phys. Chem. Chem. Phys. 2020, 22, 5604. 32100759 10.1039/c9cp06568g

[48] L. Zhang , Q. Zeng , K. Wang , J. Phys. Chem. Lett. 2017, 8, 3752. 28742359 10.1021/acs.jpclett.7b01577

[49] J. Zhao , Y. Deng , H. Wei , X. Zheng , Z. Yu , Y. Shao , J. E. Shield , J. Huang , Sci. Adv. 2017, 3, eaao5616. 29159287 10.1126/sciadv.aao5616 PMC5694650

[50] B. Murali , E. Yengel , W. Peng , Z. Chen , M. S. Alias , E. Alarousu , B. S. Ooi , V. Burlakov , A. Goriely , M. Eddaoudi , O. M. Bakr , O. F. Mohammed , J. Phys. Chem. Lett. 2017, 8, 137. 27966364 10.1021/acs.jpclett.6b02684

[51] N. Qu , Y. Lei , X. Yang , X. Hu , W. Zhao , C. Zhao , Z. Zheng , J. Mater. Chem. C 2020, 8, 8451.

[52] Q. Dong , Y. Fang , Y. Shao , P. Mulligan , J. Qiu , L. Cao , J. Huang , Science 2015, 347, 967. 25636799 10.1126/science.aaa5760

[53] S. Yang , S. Chen , E. Mosconi , Y. Fang , X. Xiao , C. Wang , Y. Zhou , Z. Yu , J. Zhao , Y. Gao , F. De Angelis , J. Huang , Science 2019, 365, 473. 31371610 10.1126/science.aax3294

[54] J. Duan , Y. Zhao , X. Yang , Y. Wang , B. He , Q. Tang , Adv. Energy Mater. 2018, 8, 1802346.

[55] Z. Liu , J. Chang , Z. Lin , L. Zhou , Z. Yang , D. Chen , C. Zhang , S. F. Liu , Y. Hao , Adv. Energy Mater. 2018, 8, 1703432.

[56] Z. Ren , J. Wang , Z. Pan , K. Zhao , H. Zhang , Y. Li , Y. Zhao , I. Mora‐Sero , J. Bisquert , X. Zhong , Chem. Mater. 2015, 27, 8398.

[57] T. Chen , G. Tong , E. Xu , H. Li , P. Li , Z. Zhu , J. Tang , Y. Qi , Y. Jiang , J. Mater. Chem. A 2019, 7, 20597.

[58] J. Liu , H.‐B. Zhang , R. Sun , Y. Liu , Z. Liu , A. Zhou , Z.‐Z. Yu , Adv. Mater. 2017, 29, 1702367. 10.1002/adma.201702367 28799671

[59] Y. Cho , H. D. Kim , J. Zheng , J. Bing , Y. Li , M. Zhang , M. A. Green , A. Wakamiya , S. Huang , H. Ohkita , A. W. Y. Ho‐Baillie , ACS Energy Lett. 2021, 6, 925.

