# Supplementary material for: Tailored Lattice “Tape” to Confine Tensile Interface for 11.08%‐Efficiency All‐Inorganic CsPbBr3 Perovskite Solar Cell with an Ultrahigh Voltage of 1.702 V
Source: Adv Sci (Weinh). 2021 Aug 8;8(19):2101418. doi: 10.1002/advs.202101418 (PMC8498907; doi:10.1002/advs.202101418)
Supplement: Supplementary file 1 — Supporting Information [file ADVS-8-2101418-s001.pdf]

© 2021 Wiley-VCH GmbH

ADVANCED SCIENCE
Open Access

Supporting Information

for *Adv. Sci.*, DOI: 10.1002/advs.202101418

Tailored Lattice "Tape" to Confine Tensile Interface for 11.08%-Efficiency All-inorganic $CsPbBr_3$ Perovskite Solar Cell with an Ultrahigh Voltage of 1.702 V

Qingwei Zhou, Jialong Duan, Jian Du, Qiyao Guo, Qiaoyu Zhang, Xiya Yang, Yanyan Duan and Qunwei Tang**

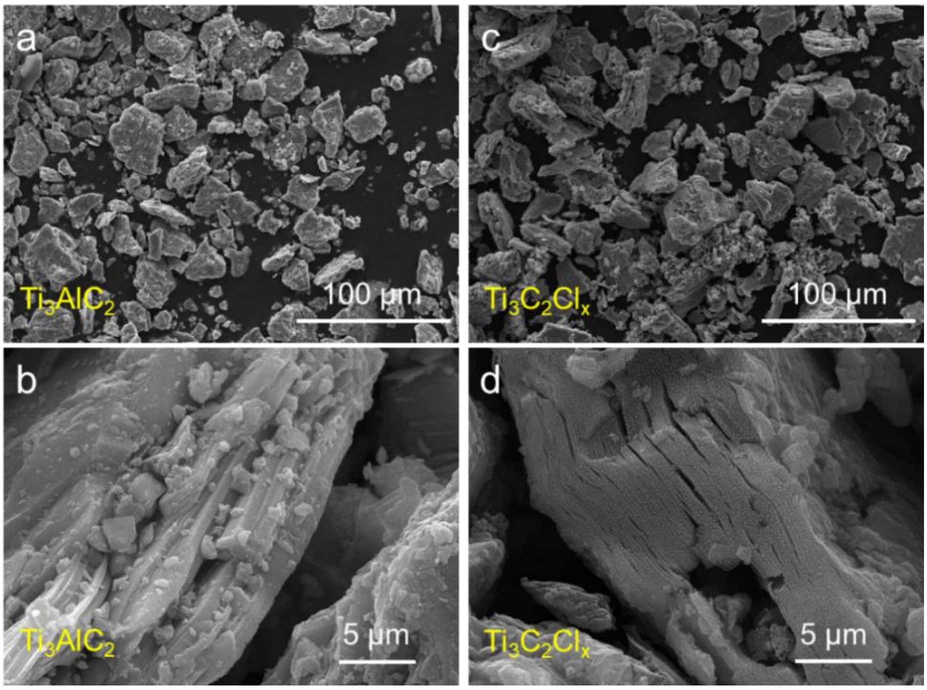

Figure S1. SEM images of (a,b) Ti_3AlC_2 MAX and (c,d) $Ti_3C_2Cl_x$ MXene.

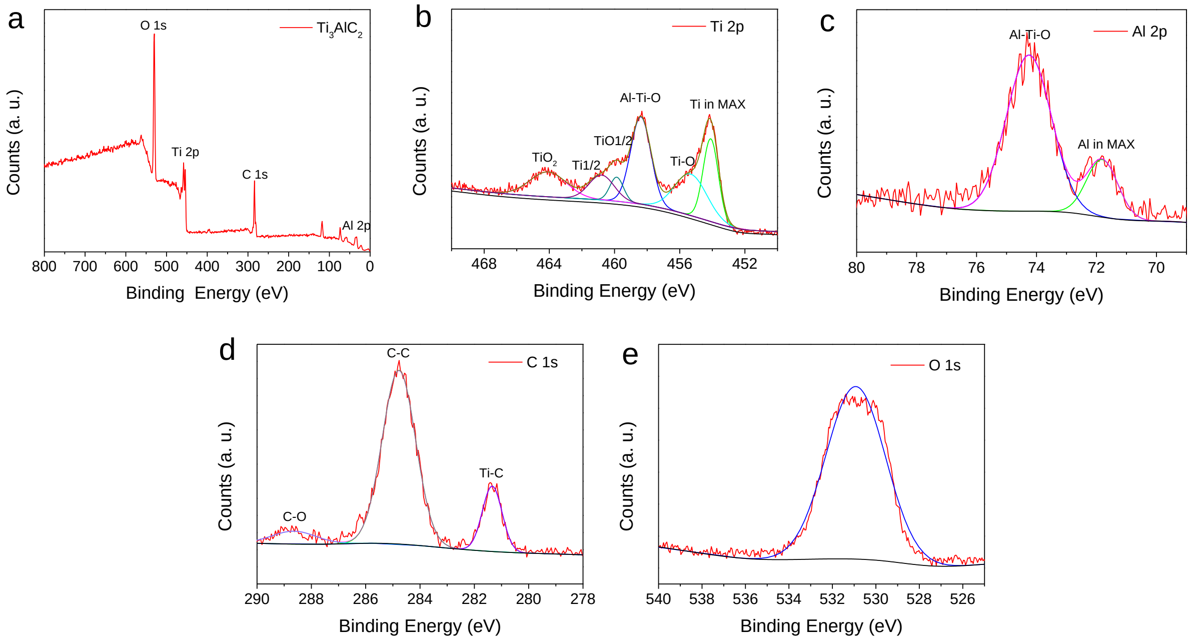

Figure S2. (a) The whole XPS spectrum of Ti_3AlC_2 powder. High-resolution XPS spectra and fitted curves of (b) Ti 2*p*, (c) Al 2*p*, (d) C 1*s* and (d) O 1*s* for Ti_3AlC_2 MAX.

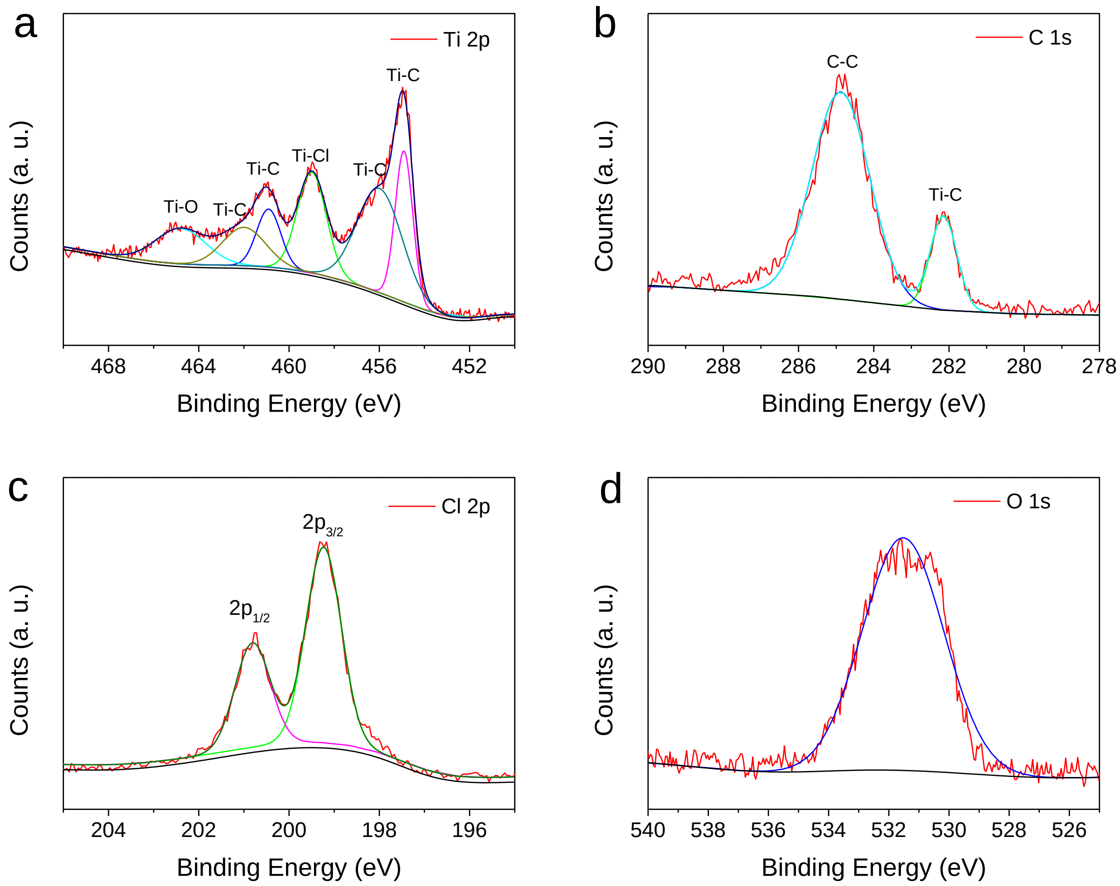

Figure S3. High-resolution XPS spectra and fitted curves of (a) Ti 2*p*, (b) C 1*s*, (c) Cl 2*p* and (d) O 1*s* for $Ti_3C_2Cl_x$ MXene.

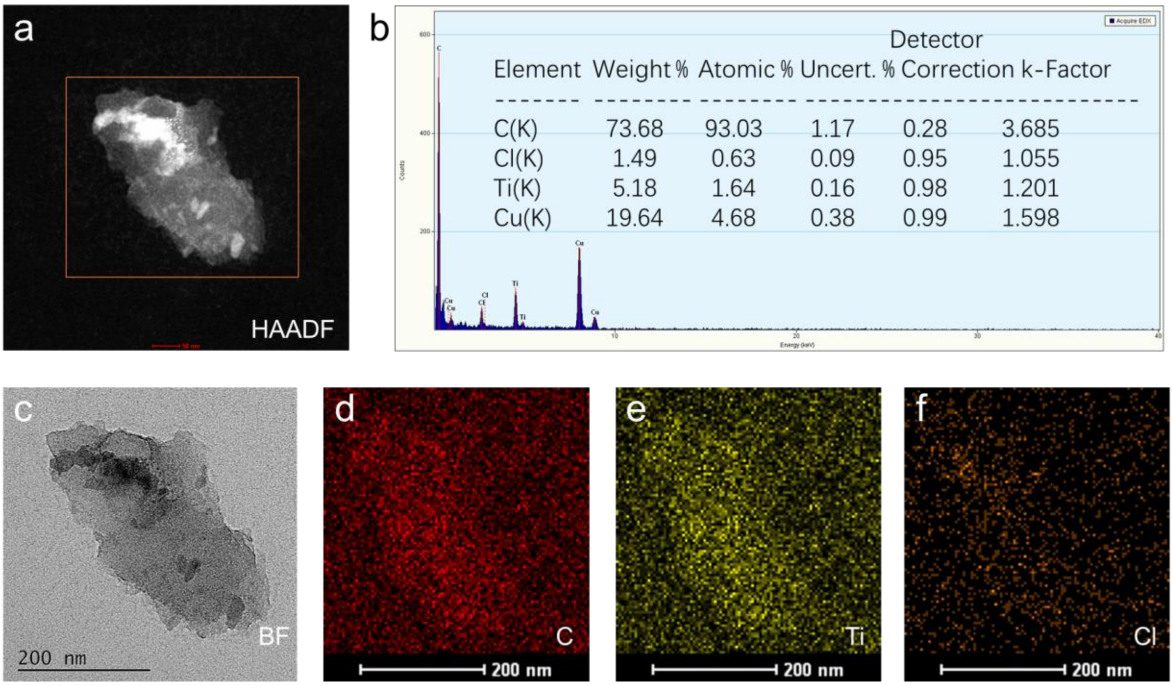

Figure S4. TEM image and the corresponding EDS mapping images of $Ti_3C_2Cl_x$ MXene.

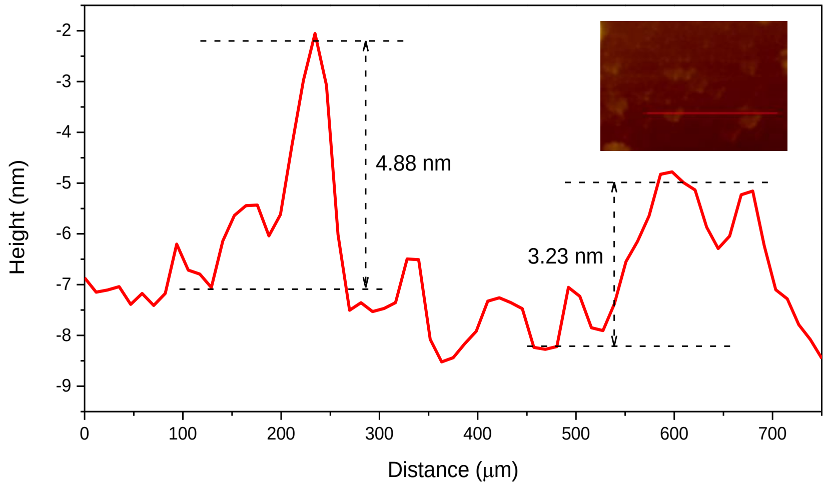

Figure S5. Height profile of $Ti_3C_2Cl_x$ MXene, insert is the corresponding AFM image.

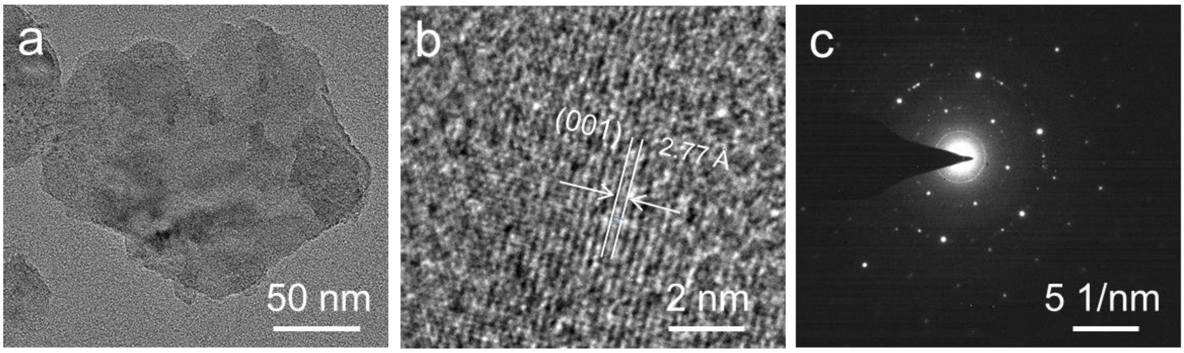

Figure S6. (a) TEM image, (b) HRTEM image and (c) the corresponding SAED pattern of the delaminated $Ti_3C_2Cl_x$ MXene.

Figure S6a shows the transmission electron microscopy (TEM) image of a typical delaminated $Ti_3C_2Cl_x$ MXene with lateral dimension of hundreds of nanometers and thickness of ~ 4 nm (Figure S5), indicating that the obtained $Ti_3C_2Cl_x$ MXenes are around three layers.[1] From the high-resolution TEM (HRTEM) image in Figure S6b and the corresponding selected area electron diffraction (SAED) pattern of $Ti_3C_2Cl_x$ MXene in Figure S6c, ordered atom matrices with clear lattice fringes and a single set of spot patterns with hexagonal distribution together verify the detrimental defect-free $Ti_3C_2Cl_x$ MXene.

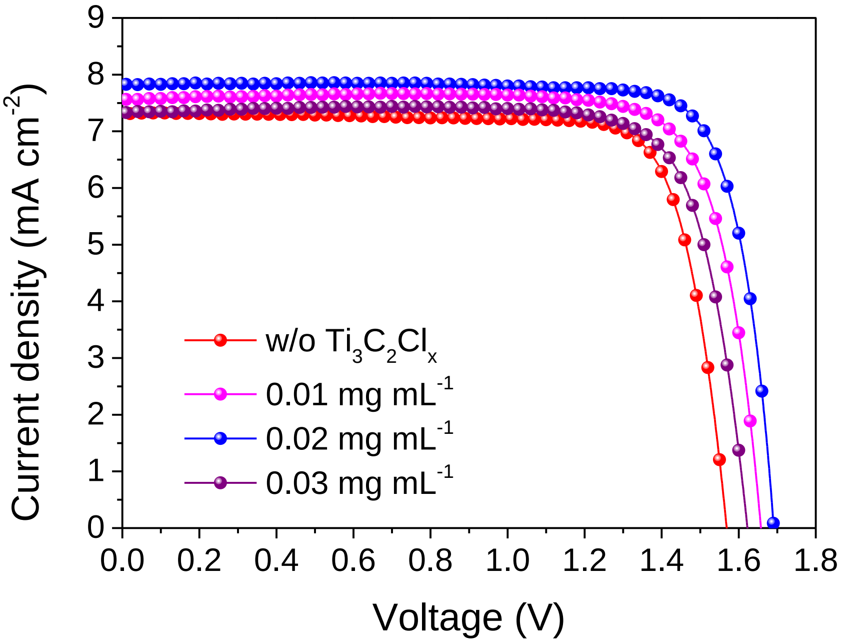

Figure S7. Reverse *J*-*V* curves of PSCs with different amounts of $Ti_3C_2Cl_x$ MXene. The corresponding photovoltaic parameters are summarized in Table S1.

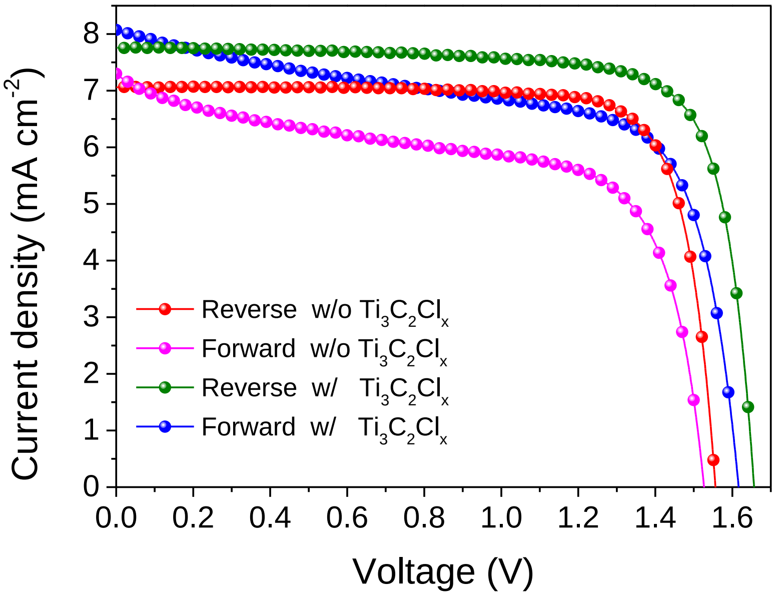

Figure S8. *J*-*V* curves for the $CsPbBr_3$ PSCs with and without $Ti_3C_2Cl_x$ additives under reverse and forward scans. The corresponding photovoltaic parameters are summarized in Table S3.

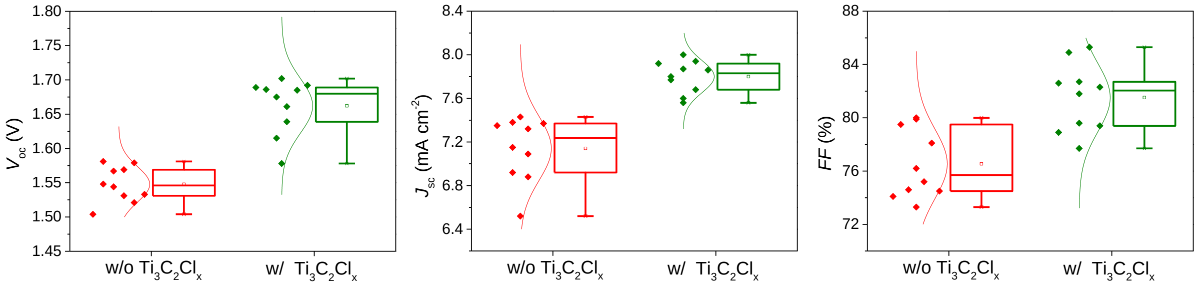

Figure S9. Statistical distributions of V_{oc}, J_{sc} and *FF* for PSCs with and without $Ti_3C_2Cl_x$ MXene.

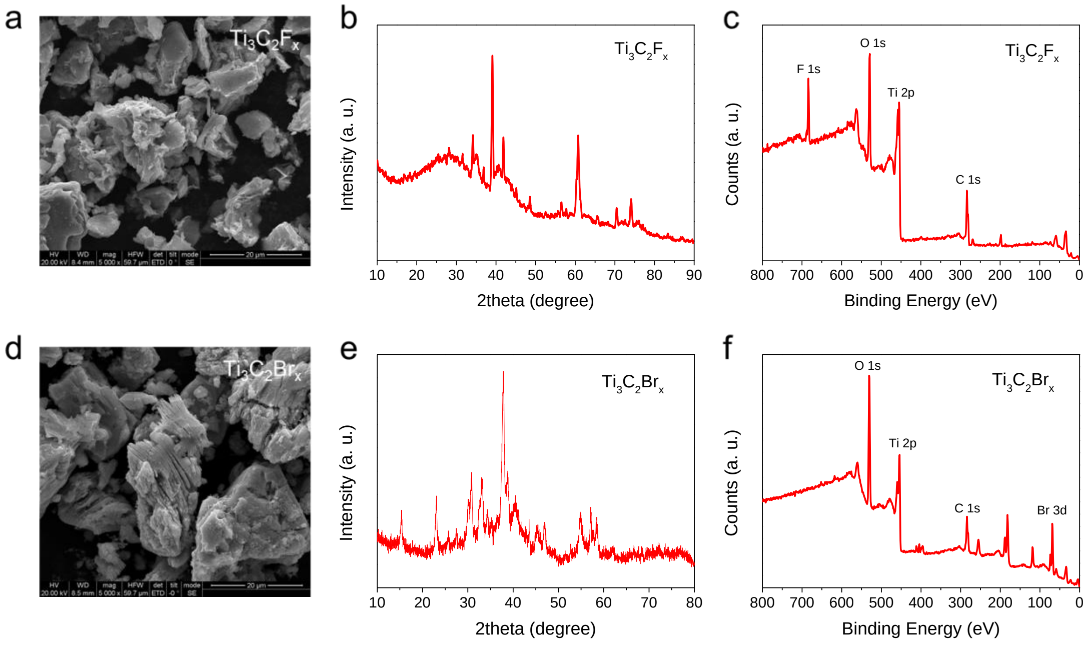

Figure S10. (a, d) SEM images, (b, e) XRD patterns and (c, f) XPS spectra of the obtained $Ti_3C_2F_x$ and $Ti_3C_2Br_x$ MXenes.

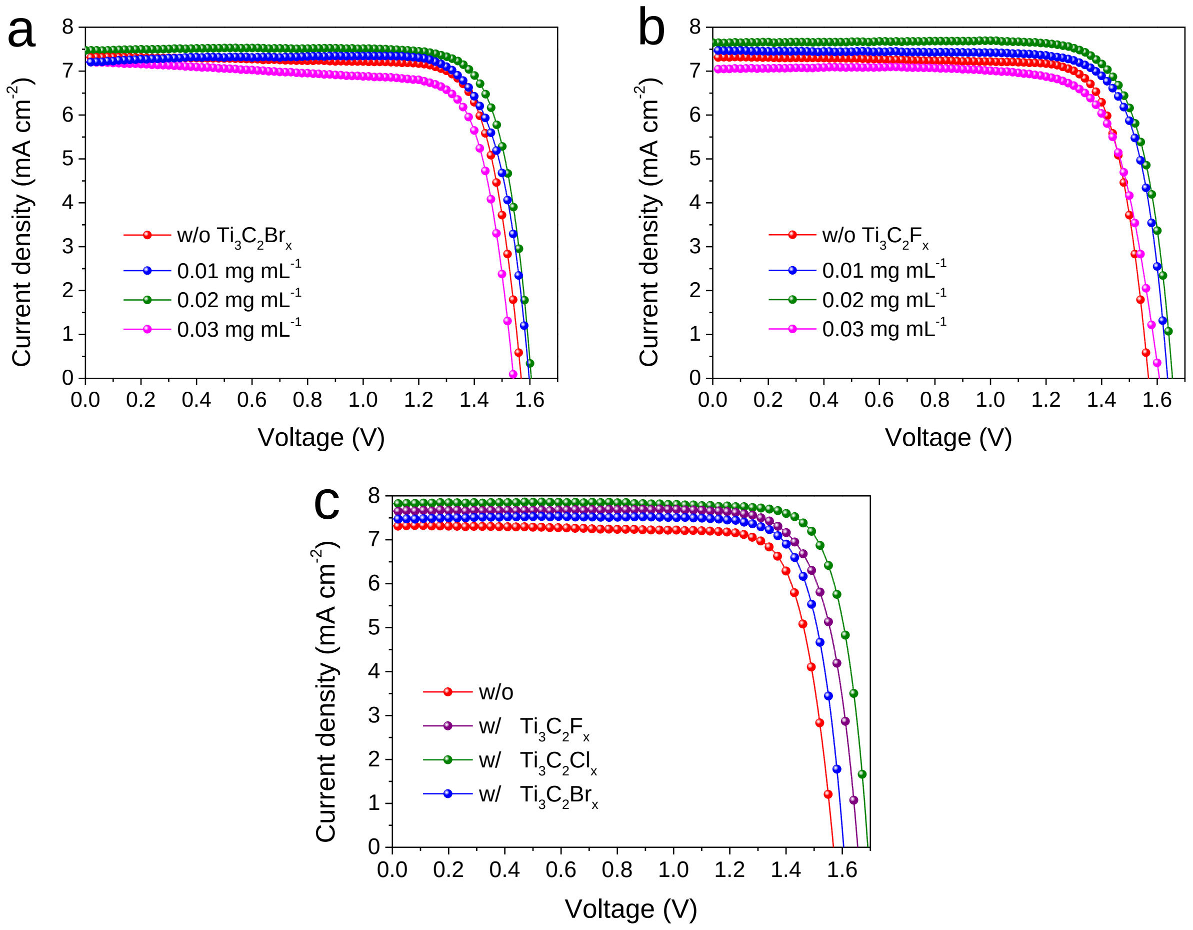

Figure S11. *J-V* curves of PSCs with different amounts of (a) $Ti_3C_2Br_x$ MXene and (b) $Ti_3C_2F_x$ MXene. The corresponding photovoltaic parameters are summarized in Table S4. (c) *J-V* curves of optimized PSC devices with various Ti_3C_2 MXene additives, in which the optimized concentration is 0.02 mg mL^{-1} for all Ti_3C_2 halide-mediated devices.

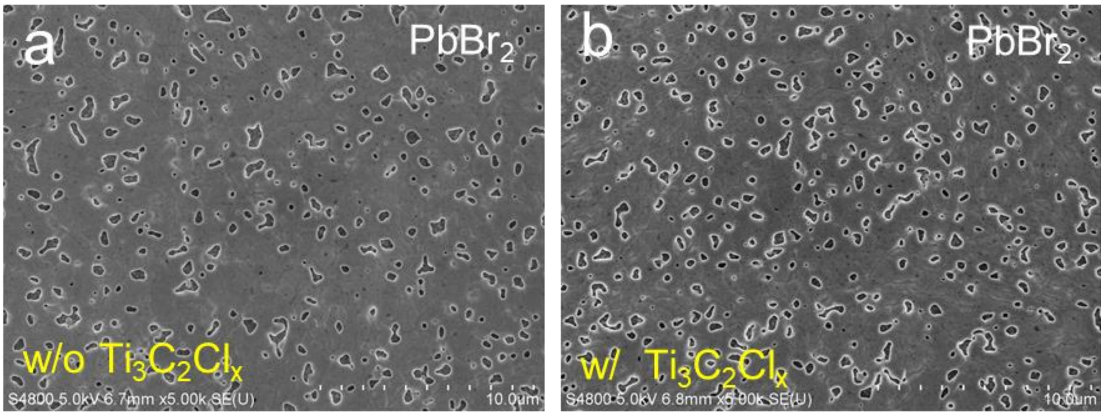

Figure S12. Top-view SEM images of $PbBr_2$ films with and without $Ti_3C_2Cl_x$ MXene.

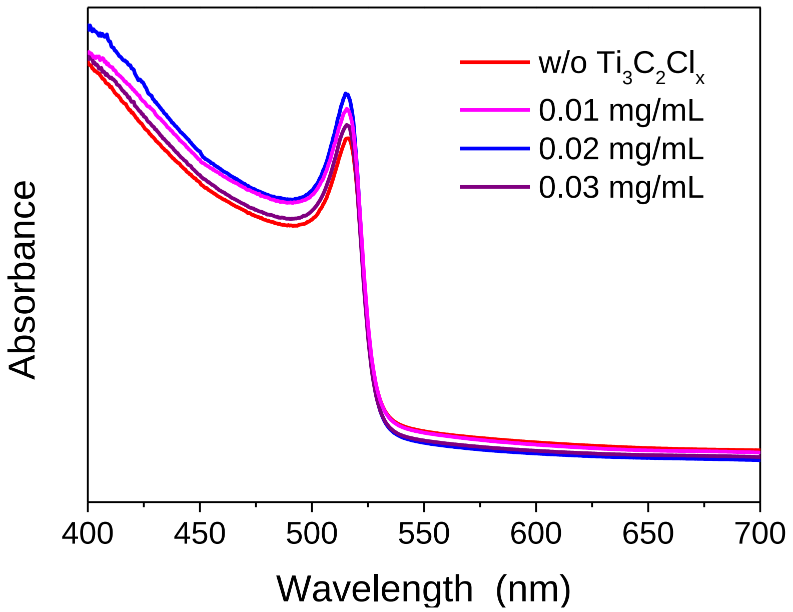

Figure S13. UV-vis spectra of $CsPbBr_3$ perovskite films with different amount of $Ti_3C_2Cl_x$ MXene in the $PbBr_2$ solution.

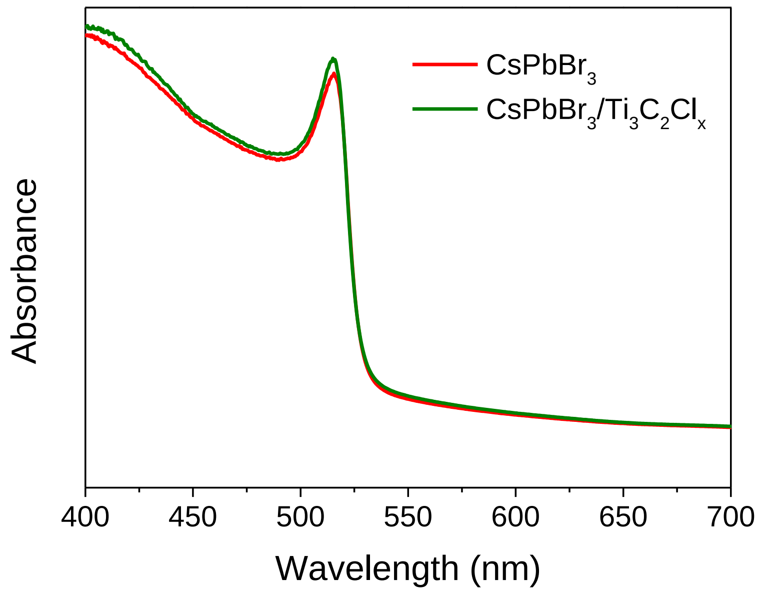

Figure S14. UV-vis spectra of $CsPbBr_3$ perovskite films with and without modification of $Ti_3C_2Cl_x$ MXene on its surface.

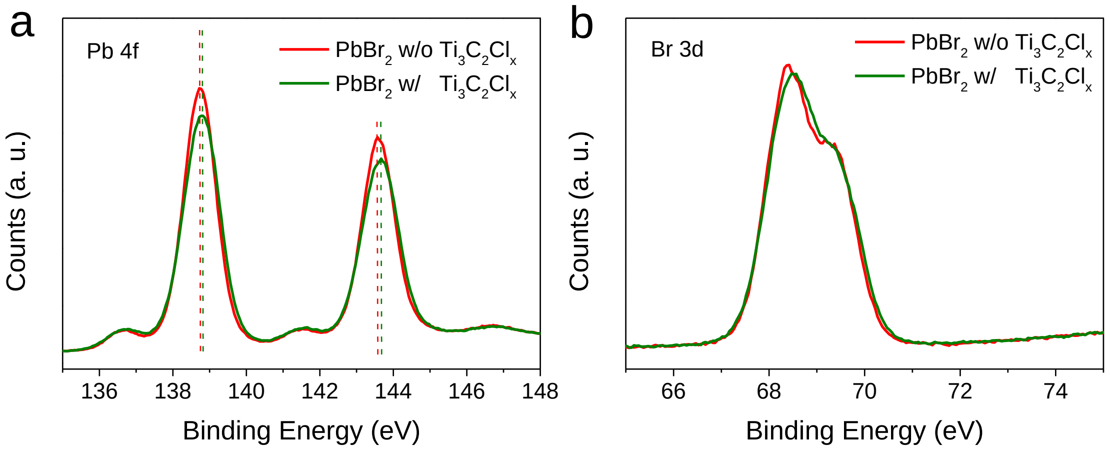

Figure S15. XPS spectra of (a) Pb 4*f* and (b) Br 3*d* for $PbBr_2$ films with and without $Ti_3C_2Cl_x$ MXene.

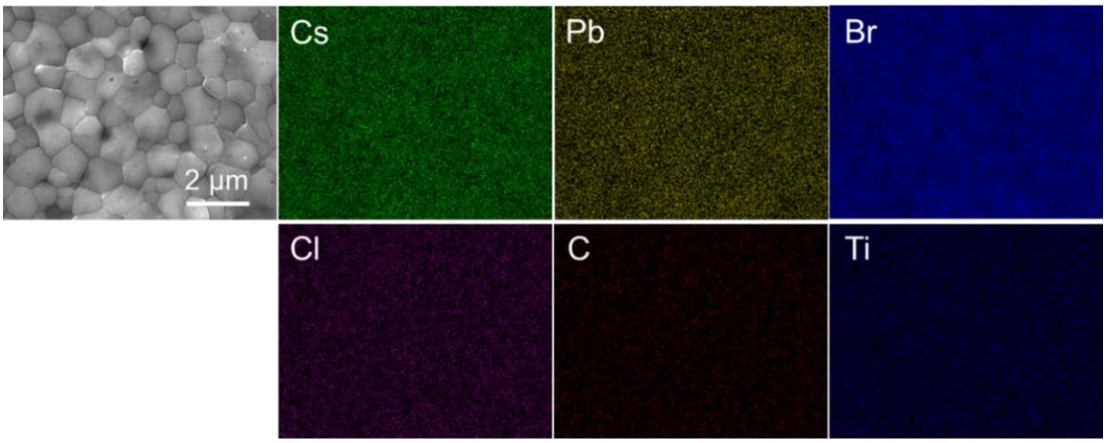

Figure S16. SEM image and the corresponding EDS mapping images of $CsPbBr_3$ perovskite film with $Ti_3C_2Cl_x$ MXene.

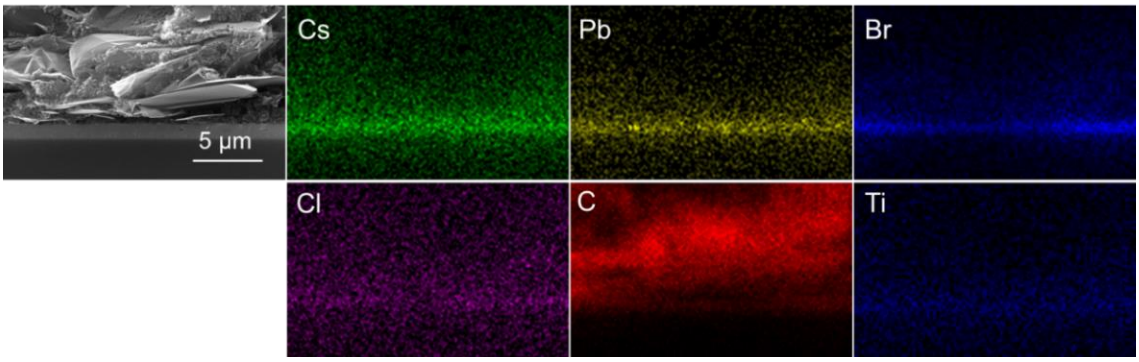

Figure S17. Cross-sectional SEM image and the corresponding EDS mapping images of PSC device with $Ti_3C_2Cl_x$ MXene.

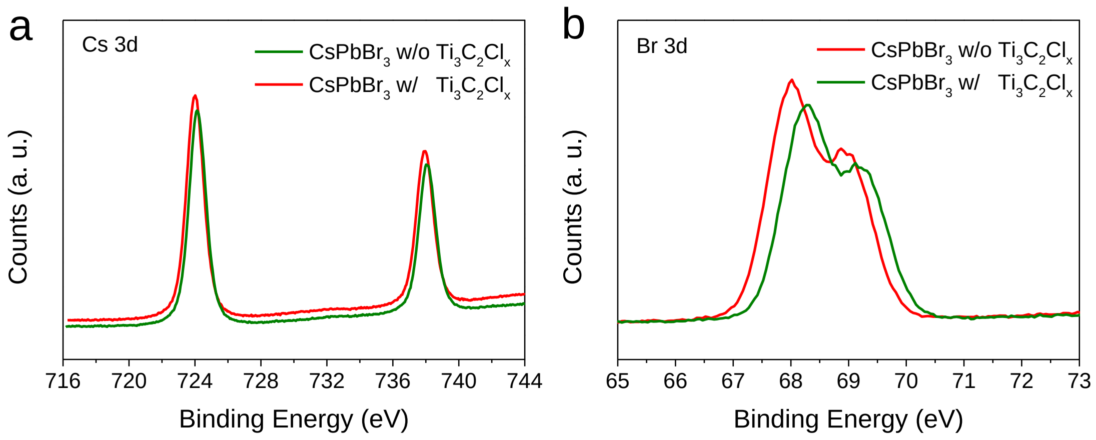

Figure S18. XPS spectra of (a) Cs 3*d* and (b) Br 3*d* for $CsPbBr_3$ perovskite films with and without $Ti_3C_2Cl_x$ MXene.

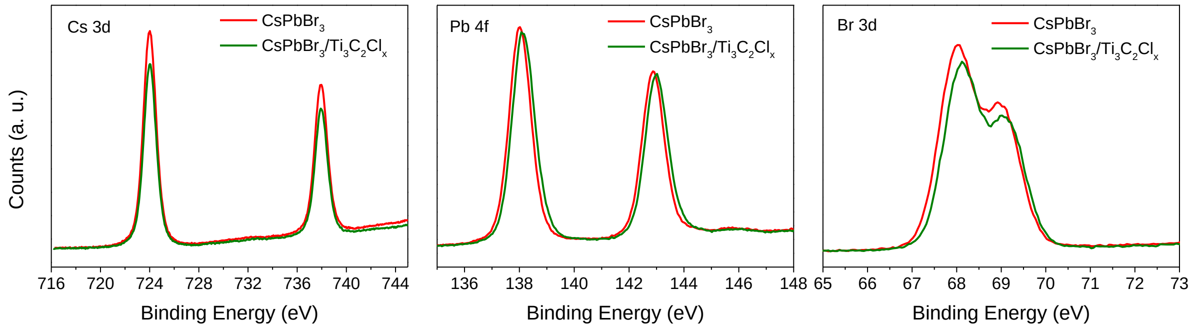

Figure S19. XPS spectra of Cs 3*d*, Pb 4*f*, Br 3*d* for $CsPbBr_3$ films with and without surface modification by $Ti_3C_2Cl_x$ MXene.

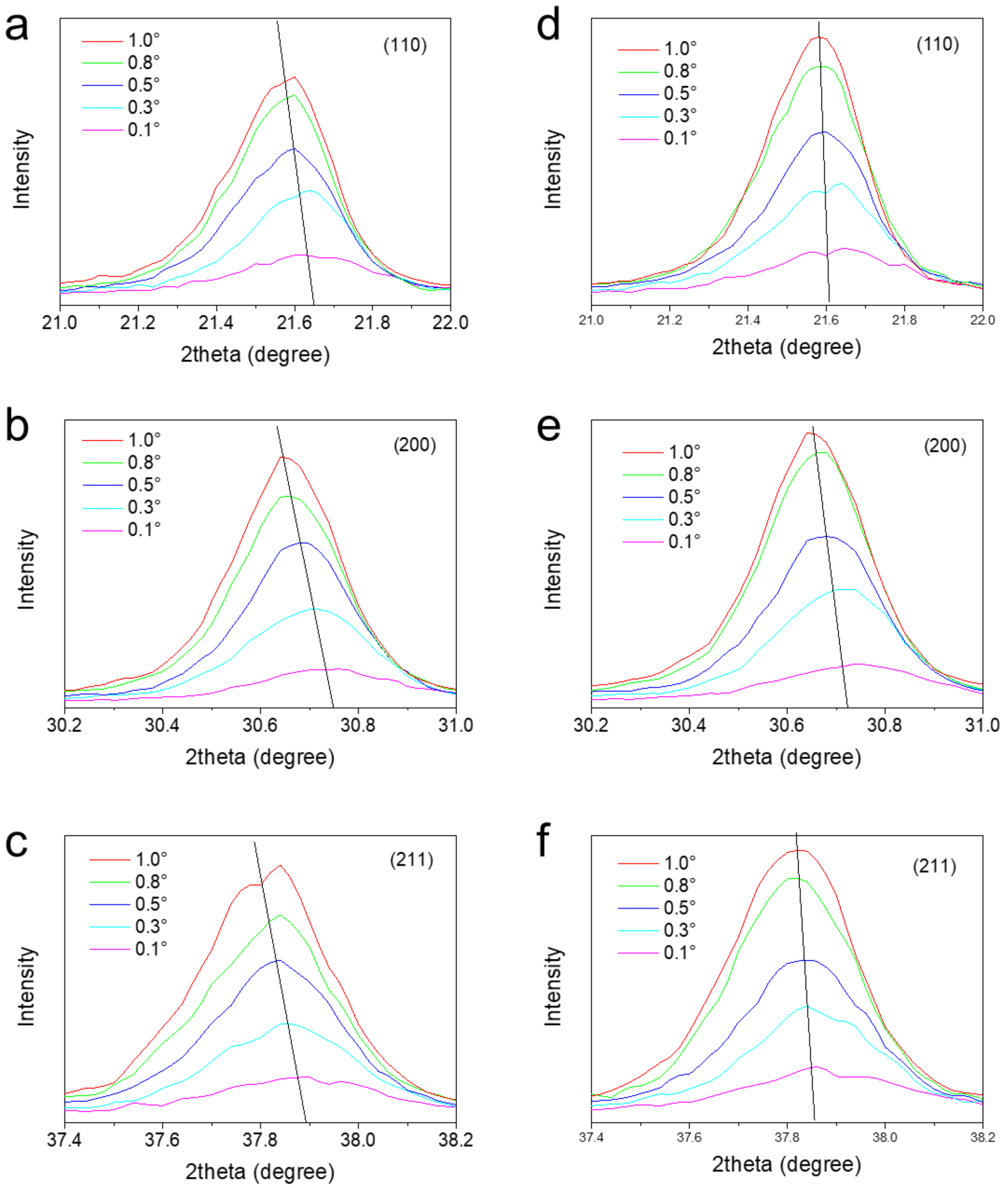

Figure S20. GIXRD patterns of the $CsPbBr_3$ perovskite films (a, b and c) with and (d, e and f) without $Ti_3C_2Cl_x$ modification.

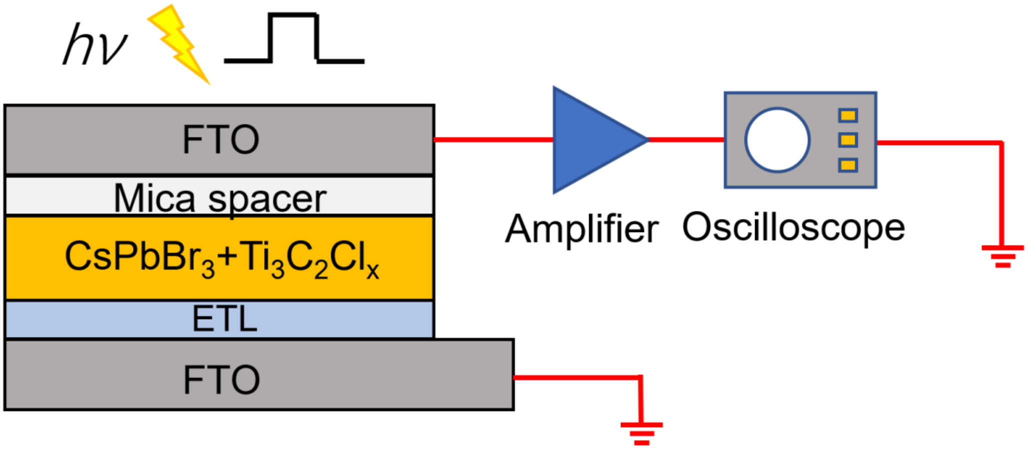

Figure S21. A schematic setup for TSPV characterization.

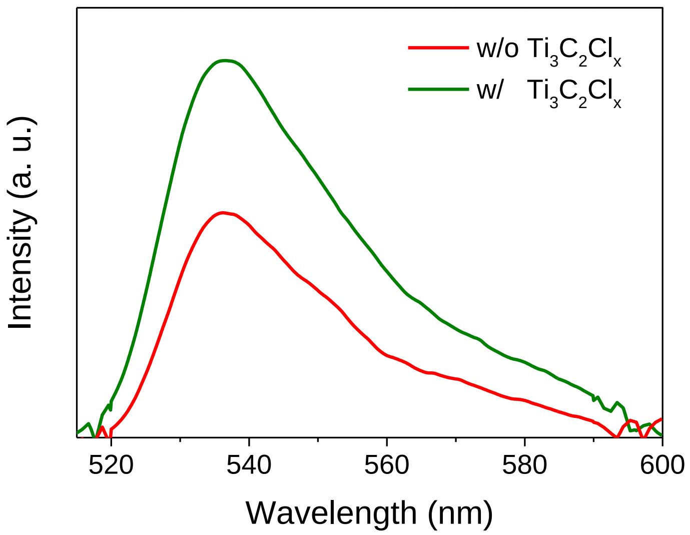

Figure S22. PL spectra of $CsPbBr_3$ films with and without $Ti_3C_2Cl_x$ MXene.

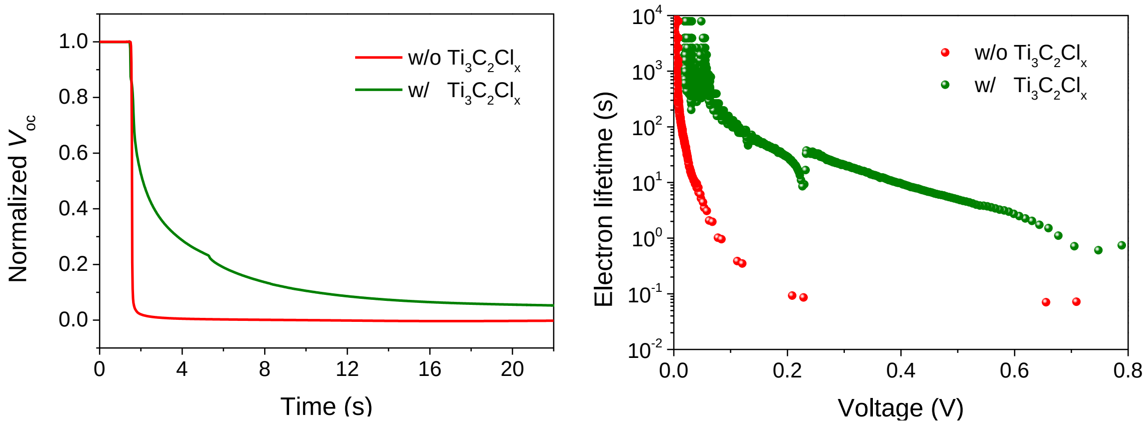

Figure S23. V_{oc} decay curves (left) and the calculated electron lifetime (right) from V_{oc} decay curves for the $CsPbBr_3$ PSCs with and without $Ti_3C_2Cl_x$ MXene.

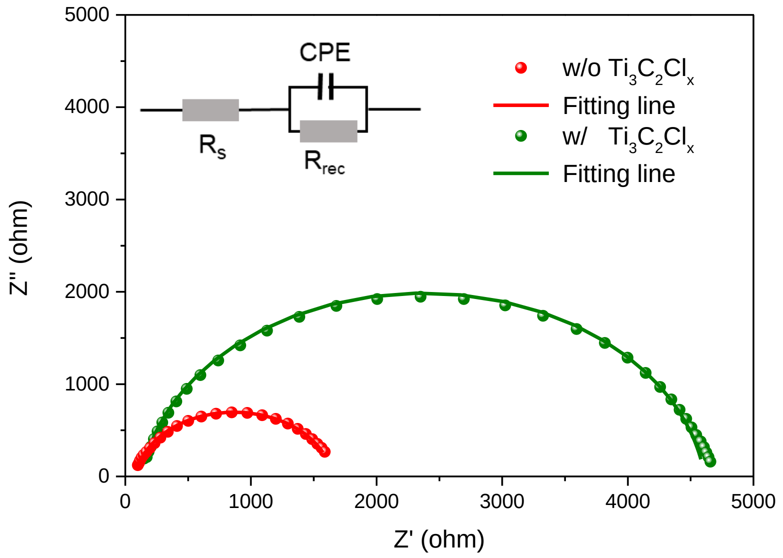

Figure S24. EIS plots of the $CsPbBr_3$ PSCs with and without $Ti_3C_2Cl_x$ MXene.

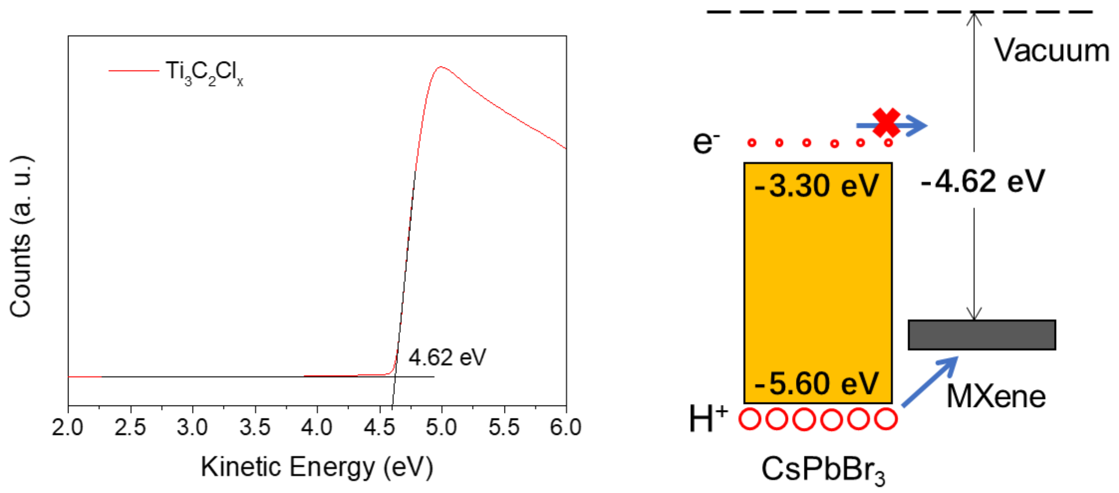

Figure S25. Ultraviolet photoelectron spectrum of $Ti_3C_2Cl_x$, and the energy level alignment between $Ti_3C_2Cl_x$ MXene and $CsPbBr_3$ perovskite.

Table S1 Photovoltaic parameters for PSCs with different amounts of $Ti_3C_2Cl_x$ MXene.

	V_{oc} (V)	J_{sc} (mA cm^{-2})	PCE (%)	*FF* (%)
w/o $Ti_3C_2Cl_x$	1.569	7.32	9.18	79.9
0.01 mg mL^{-1}	1.658	7.56	10.03	80.0
0.02 mg mL^{-1}	1.691	7.82	10.80	81.7
0.03 mg mL^{-1}	1.623	7.34	9.45	79.3

Table S2 Comparison of photovoltaic parameters for state-of-the-art $CsPbBr_3$ PSCs.

Devices	V_{oc} (V)	J_{sc} (mA cm^{-2})	PCE (%)	*FF* (%)	Ref.
**FTO/SnO_2-TiO_xCl_{4-2x}/$CsPbBr_3$+$Ti_3C_2Cl_x$/$Ti_3C_2Cl_x$/Carbon**	**1.702**	**7.87**	**11.08**	**82.7**	**This work**
FTO/SnO_2-TiO_xCl_{4-2x}/WS_2/$CsPbBr_3$/Carbon	1.70	7.95	10.65	79	S[2]
FTO/SnO_2/$CsPbBr_3$/$CsSnBr_3$/Carbon	1.610	7.80	10.60	84.4	S[3]
FTO/SnO_2/TiO_xCl_{4-2x}/Rb^+-$CsPbBr_3$/Carbon	1.629	7.96	10.44	80.5	S[4]
FTO/Sb-TiO_2/$CsPbBr_3$/C	1.654	6.70	8.91	80.4	S[5]
FTO/*L*-TiO_2:$MoSe_2$/$CsPbBr_3$/C	1.615	7.88	10.02	78.7	S[6]
FTO/*c*-TiO_2/*m*-TiO_2/$CsPbBr_3$/PTAA/Au	1.25	6.70	6.20	73.0	S[7]
FTO/*c*-TiO_2/*m*-TiO_2/$CsPbBr_3$/C	1.29	5.70	5.00	68.0	S[8]
FTO/ZnO/$CsPbBr_3$-$CsPb_2Br_5$/Spiro-OMeTAD/Au	1.43	6.17	6.81	77.2	S[9]
FTO/*c*-TiO_2/*m*-TiO_2/$CsPbBr_3$/Spiro-OMeTAD/Au	1.34	6.52	6.05	69.0	S[10]
ITO/ZnO/$CsPbBr_3$/Spiro-OMeTAD/Au	1.38	6.15	5.98	70.51	S[11]
FTO/TiO_2/CQD-$CsPbBr_3$ IO/Spiro-OMeTAD/Au	1.06	11.34	8.29	69.0	S[12]
FTO/*c*-TiO_2/*m*-TiO_2/$CsPbBr_3$/C	1.24	7.4	6.7	73.0	S[13]
FTO/*c*-TiO_2/$CsPbBr_3$/C	1.34	6.46	5.86	68.04	S[14]
FTO/*m*-TiO_2/$CsPbBr_3$/PTAA/Au	1.27	6.16	5.72	73	S[15]
FTO/*c*-TiO_2/*m*-TiO_2/GQDs/$CsPbBr_3$/C	1.458	8.12	9.72	82.1	S[16]
FTO/*c*-TiO_2/*m*-TiO_2/Sm^{3+}-$CsPbBr_3$/C	1.594	7.48	10.14	85.1	S[17]
FTO/SnO_2/$CsPbBr_3$/N-CQDs/C	1.622	7.87	10.71	80.1	S[18]
FTO/*c*-TiO_2/*m*-TiO_2/Sm^{3+}-$CsPbBr_3$/Cu(Cr,Ba)O_2/C	1.615	7.81	10.79	85.5	S[19]
FTO/*c*-TiO_2/*m*-TiO_2/$CsPbBr_3$/$CuInS_2$/ZnS QDs/LPP-C	1.626	7.73	10.85	86.3	S[20]
FTO/*c*-TiO_2/*m*-TiO_2/Sr^{2+}-$CsPbBr_3$/C	1.54	7.71	9.63	81.1	S[21]
FTO/*c*-TiO_2/*m*-TiO_2/Rb^+-$CsPbBr_3$/C	1.552	7.73	9.86	82.2	S[22]
FTO/*c*-TiO_2/*m*-TiO_2/GQDs/$CsPbBr_3$/MnS/C	1.52	8.28	10.45	83	S[23]
FTO/*c*-TiO_2/*m*-TiO_2/$CsPbBr_3$/Spiro-OMeTAD/Ag	1.37	6.41	6.32	72	S[24]
FTO/TiO_2/$CsPbBr_3$/C	1.19	7.48	6.12	68.8	S[25]
FTO/*c*-TiO_2/$CsPbBr_3$/C	1.49	6.89	8.11	79	S[26]
FTO/*c*-TiO_2/PTI-$CsPbBr_3$/spiro-OMeTAD/Ag	1.498	9.78	10.91	74.47	S[27]
FTO/*c*-TiO_2/*m*-TiO_2/GQDs/$CsPbBr_3$/P3HT/C	1.36	7.02	6.49	68	S[28]
FTO/*c*-TiO_2/SnO_2/$CsPbBr_3$/CuPc/C	1.31	8.24	8.79	81.4	S[29]
FTO/*c*-TiO_2/$CsPbBr_3$/C	1.545	7.37	9.35	82.2	S[30]
FTO/*c*-TiO_2/$CsPbBr_3$/Ti_3C_2-MXene/C	1.444	8.54	9.01	73.08	S[31]
FTO/*c*-TiO_2/*m*-TiO_2/Sn^{2+}-$CsPbBr_3$/C	1.37	7.66	8.63	82.22	S[32]
FTO/*c*-TiO_2/*m*-TiO_2/$CsPbBr_3$/C	1.22	7.40	7.37	84.1	S[33]
FTO/TiO_2/$CsPb_{0.998}Co_{0.002}Br_3$/Spiro-OMeTAD/Au	1.357	7.45	8.57	84.84	S[34]
FTO/*c*-TiO_2/$CsPbBr_3$/$CsPbBr_3$-$CsPb_2Br_5$/$CsPbBr_3$-Cs_4PbBr_6/C	1.461	9.26	10.17	75.39	S[35]
FTO/*c*-TiO_2/$CsPbBr_3$/spiro-OMeTAD/Au	1.27	6.97	6.95	78.5	S[36]
FTO/*c*-TiO_2/*m*-TiO_2/*m*-ZrO_2/$CsPbBr_3$/m-carbon	1.44	7.75	8.2	73.52	S[37]
FTO/*c*-TiO_2/$CsPbBr_3$–$CsPb_2Br_5$/spiro-OMeTAD/Ag	1.296	8.48	8.34	75.9	S[38]
FTO/*c*-TiO_2/$CsPbBr_3$/spiro-OMeTAD/Au	1.5	5.6	5.4	62	S[39]

Table S3 Photovoltaic parameters for PSCs with and without $Ti_3C_2Cl_x$ under reverse and forward scans.

Device	V_{oc} (V)	J_{sc} (mA cm^{-2})	PCE (%)	*FF* (%)
Reverse w/o $Ti_3C_2Cl_x$	1.556	7.06	8.72	79.3
Forward w/o $Ti_3C_2Cl_x$	1.526	7.30	6.84	60.0
Reverse w/ $Ti_3C_2Cl_x$	1.656	7.75	10.02	78.1
Forward w/ $Ti_3C_2Cl_x$	1.616	8.07	8.53	65.4

Table S4 Photovoltaic parameters for PSCs with different amounts of $Ti_3C_2Br_x$ or $Ti_3C_2F_x$ MXene.

Device	V_{oc} (V)	J_{sc} (mA cm^{-2})	PCE (%)	*FF* (%)
w/o Ti_3C_2 MXene	1.569	7.32	9.18	79.9
0.01 mg mL^{-1} $Ti_3C_2Br_x$	1.598	7.21	9.29	80.6
0.02 mg mL^{-1} $Ti_3C_2Br_x$	1.605	7.47	9.73	81.2
0.03 mg mL^{-1} $Ti_3C_2Br_x$	1.542	7.22	8.57	77.0
0.01 mg mL^{-1} $Ti_3C_2F_x$	1.637	7.46	9.65	79.0
0.02 mg mL^{-1} $Ti_3C_2F_x$	1.654	7.65	10.05	79.4
0.03 mg mL^{-1} $Ti_3C_2F_x$	1.608	7.04	8.73	77.1

Table S5. TRPL decay parameters of PSCs with and without $Ti_3C_2Cl_x$ nanosheets.

Device	τ_1 (ns)	a_1	τ_2 (ns)	a_2	τ_{ave} (ns)
w/o $Ti_3C_2Cl_x$	0.411	37.19%	34.323	62.81%	1.083
w/ $Ti_3C_2Cl_x$	0.735	30.43%	14.855	69.57%	2.169

References

[1] V. Kamysbayev, A. S. Filatov, H. Hu, X. Rui, F. Lagunas, D. Wang, R. F. Klie, D. V. Talapin, *Science* **2020**, 369, 979–983.

[2] Q. Zhou, J. Duan, X. Yang, Y. Duan, Q. Tang, *Angew. Chem. Int. Ed.* **2020**, 132, 22181–22185.

[3] Y. Zhao, J. Duan, H. Yuan, Y. Wang, X. Yang, B. He, Q. Tang, *Sol. RRL* **2019**, 1800284.

[4] Q. Zhou, J. Duan, Y. Wang, X. Yang, Q. Tang, *J. Energy Chem.* **2020**, *50*, 1–8.

[5] Y. Xu, J. Duan, X. Yang, J. Du, Y. Wang, Y. Duan, Q. Tang, *J. Mater. Chem. A* **2020**, *8*, 11859–11866.

[6] Q. Zhou, J. Du, J. Duan, Y. Wang, X. Yang, Y. Duan, Q. Tang, *J. Mater. Chem. A* **2020**, *8*, 7784–7791.

[7] M. Kulbak, S. Gupta, N. Kedem, I. Levine, T. Bendikov, G. Hodes, D. Cahen, *J. Phys. Chem. Lett.* **2016**, *7*, 167–172.

[8] X. Chang, W. Li, L. Zhu, H. Liu, H. Geng, S. Xiang, J. Liu, H. Chen, *ACS Appl. Mater. Interfaces* **2016**, *8*, 33649–33655.

[9] X. Zhang, Z. Jin, J. Zhang, D. Bai, H. Bian, K. Wang, J. Sun, Q. Wang, S. F. Liu, *ACS Appl. Mater. Interfaces* **2018**, *10*, 7145–7154.

[10] K. C. Tang, P. You, F. Yan, *Sol. RRL* **2018**, *2*, 1800075.

[11] W. Chen, J. Zhang, G. Xu, R. Xue, Y. Li, Y. Zhou, J. Hou, Y. Li, *Adv. Mater.* **2018**, *30*, 1800855.

[12] S. Zhou, R. Tang, L. Yin, *Adv. Mater.* **2017**, *29*, 1703682.

[13] J. Liang, C. Wang, Y. Wang, Z. Xu, Z. Lu, Y. Ma, H. Zhu, Y. Hu, C. Xiao, X. Yi, G. Zhu, H. Lv, L. Ma, T. Chen, Z. Tie, Z. Jin, J. Liu, *J. Am. Chem. Soc.* **2016**, *138*, 15829–15832.

[14] P. Teng, X. Han, J. Li, Y. Xu, L. Kang, Y. Wang, Y. Yang, T. Yu, *ACS Appl. Mater. Interfaces* **2018**, *10*, 9541–9546.

[15] M. Kulbak, D. Cahen, G. Hodes, *J. Phys. Chem. Lett.* **2015**, *6*, 2452–2456.

[16] J. Duan, Y. Zhao, B. He, Q. Tang, *Angew. Chem. Int. Ed.* **2018**, *57*, 3787–3791.

[17] J. Duan, Y. Zhao, X. Yang, Y. Wang, B. He, Q. Tang, *Adv. Energy Mater.* **2018**, *8*, 1802346.

[18] Y. Zhao, J. Duan, Y. Wang, X. Yang, Q. Tang, *Nano Energy* **2020**, *67*, 104286.

[19] J. Duan, Y. Zhao, Y. Wang, X. Yang, Q. Tang, *Angew. Chem. Int. Ed.* **2019**, *58*, 16147–16151.

[20] J. Duan, Y. Wang, X. Yang, Q. Tang, *Angew. Chem. Int. Ed.* **2020**, *59*, 4391–4395.
[21] Y. Zhao, Y. Wang, J. Duan, X. Yang, Q. Tang, *J. Mater. Chem. A* **2019**, *7*, 6877–6882.
[22] Y. Li, J. Duan, H. Yuan, Y. Zhao, B. He, Q. Tang, *Sol. RRL* **2018**, *2*, 1800164.
[23] X. Li, Y. Tan, H. Lai, S. Li, Y. Chen, S. Li, P. Xu, J. Yang, *ACS Appl. Mater. Interfaces* **2019**, *11*, 29746–29752.
[24] H. Wang, Y. Wu, M. Ma, S. Dong, Q. Li, J. Du, H. Zhang, Q. Xu, *ACS Appl. Energy Mater.* **2019**, *2*, 2305–2312.
[25] X. Cao, G. Zhang, L. Jiang, Y. Cai, Y. Gao, W. Yang, X. He, Q. Zeng, G. Xing, Y. Jia, J. Wei, *ACS Appl. Mater. Interfaces* **2019**, *12*, 5925–5931.
[26] X. Wan, Z. Yu, W. Tian, F. Huang, S. Jin, X. Yang, Y.-B. Cheng, A. Hagfeldt, L. Sun, *J. Energy Chem.* **2020**, *46*, 8–15.
[27] G. Tong, T. Chen, H. Li, L. Qiu, Z. Liu, Y. Dang, W. Song, L. K. Ono, Y. Jiang, Y. Qi, *Nano Energy* **2019**, *65*, 104015.
[28] G. Wang, W. Dong, A. Gurung, K. Chen, F. Wu, Q. He, R. Pathak, Q. Qiao, *J. Power Sources* **2019**, *432*, 48–54.
[29] X. Liu, X. Tan, Z. Liu, H. Ye, B. Sun, T. Shi, Z. Tang, G. Liao, *Nano Energy* **2019**, *56*, 184–195.
[30] T. Xiang, Y. Zhang, H. Wu, J. Li, L. Yang, K. Wang, J. Xia, Z. Deng, J. Xiao, W. Li, Z. Ku, F. Huang, J. Zhong, Y. Peng, Y.-B. Cheng, *Sol. Energy Mater. Sol. Cells* **2019**, 110317.
[31] T. Chen, G. Tong, E. Xu, H. Li, P. Li, Z. Zhu, J. Tang, Y. Qi, Y. Jiang, *J. Mater. Chem. A* **2019**, *7*, 20597–20603.
[32] H. Guo, Y. Pei, J. Zhang, C. Cai, K. Zhou, Y. Zhu, *J. Mater. Chem. C* **2019**, *7*, 11234–11243.
[33] D. Huang, P. Xie, Z. Pan, H. Rao, X. Zhong, *J. Mater. Chem. A* **2019**, *7*, 22420–22428.
[34] D. Wang, W. Li, Z. Du, G. Li, W. Sun, J. Wu, Z. Lan, *J. Mater. Chem. C* **2020**, *8*, 1649–1655.
[35] G. Tong, T. Chen, H. Li, W. Song, Y. Chang, J. Liu, L. Yu, J. Xu, Y. Qi, Y. Jiang, *Sol. RRL* **2019**, *3*, 1900030.
[36] J. Lei, F. Gao, H. Wang, J. Li, J. Jiang, X. Wu, R. Gao, Z. Yang, S. (Frank) Liu, *Sol. Energy Mater. Sol. Cells* **2018**, *187*, 1–8.
[37] I. Poli, J. Baker, J. McGettrick, F. De Rossi, S. Eslava, T. Watson, P. J. Cameron, *J. Mater. Chem. A* **2018**, *6*, 18677–18686.

[38] H. Li, G. Tong, T. Chen, H. Zhu, G. Li, Y. Chang, L. Wang, Y. Jiang, *J. Mater. Chem. A* **2018**, *6*, 14255–14261.

[39] Q. A. Akkerman, M. Gandini, F. Di Stasio, P. Rastogi, F. Palazon, G. Bertoni, J. M. Ball, M. Prato, A. Petrozza, L. Manna, *Nat. Energy* **2017**, *2*, 16194.
